# Supplementary material for: Risk factors associated with post-acute sequelae of SARS-CoV-2: an N3C and NIH RECOVER study
Source: BMC Public Health. 2023 Oct 25;23:2103. doi: 10.1186/s12889-023-16916-w (PMC10601201; doi:10.1186/s12889-023-16916-w)
Supplement: Supplementary file 1 — Additional file 1. eMethods, eResults, eFigures, eTables, eMethods, Data, eResults, Sensitivity Analysis: Other Definitions of PASC. [file 12889_2023_16916_MOESM1_ESM.docx]

**Supplementary Material**

Hill EL, Mehta HB, Sharma S., et al. **Risk Factors Associated with PASC in an EHR Cohort: A National COVID Cohort Collaborative (N3C) Analysis as part of the NIH RECOVER program**

**eMethods**

**eResults**

**eFigures**

eFigure 1. Feature Importance XGBoost (PASC defined as U09.9 or Long-COVID Clinic Visit)

eFigure 2. Forest Plot for Unrestricted Controls without SDoH (PASC defined as U09.9 or Long-COVID Clinic Visit)

eFigure 3. Forest Plot for Age association (Male)

eFigure 4. Forest Plot for Age association (Female)

eFigure 5. Feature Importance XGBoost for Unrestricted Controls with SDoH (PASC defined as U09.9 or Long-COVID Clinic Visit)

eFigure 6. Feature Importance XGBoost for Unrestricted Controls with SDoH–Hospitalized Sample (PASC defined as U09.9 or Long COVID Visit)

eFigure 7. Feature Importance XGBoost for Unrestricted Controls with SDoH –non-Hospitalized Sample (PASC defined as U09.9 or Long COVID Visit)

**eTables**

eTable 1. Cohort Characteristics for PASC cases defined by U09.9 or clinic visit (Additional Characteristics Not Shown in Table 1)

eTable 2. PASC Risk Factors from Logistic Regression (PASC defined as U09.9 or long-COVID Clinic Visit)

eTable 3. AUC scores for our three models of PASC defined by U09.9 or Long-COVID Clinic Visit

eTable 4. Comparison of Feature Importance Across Models for Unrestricted Sample (PASC defined as U09.9 or Long-COVID Clinic Visit)

eTable 5. Comparison of Feature Importance Across Models for Restricted Sample (PASC defined as U09.9 or Long-COVID Clinic Visit)

eTable 6. Comparison of Feature Importance Across Models for More Restricted Sample (PASC defined as U09.9 or Long-COVID Clinic Visit)

eTable 7. Comparison of Feature Importance Across Models for Unrestricted Sample including SDOH variables (PASC defined as U09.9 or Long-COVID Clinic Visit)

eTable 8. Cohort Characteristics for PASC cases defined by U09.9 or clinic visit for Hospitalized During COVID Index

eTable 9. Cohort Characteristics for PASC cases defined by U09.9 or clinic visit for Not Hospitalized During COVID Index11

eTable 10. PASC Risk Factors from Logistic Regression including SDoH (PASC defined as U09.9 or long-COVID visit) Not Hospitalized during COVID Index

eTable 11. PASC Risk Factors from Logistic Regression including SDoH (PASC defined as U09.9 or long-COVID visit) Hospitalized during COVID Index

eTable 12. Comparison of Feature Importance Across Models for Hospitalized during Index COVID for Unrestricted Sample including SDOH variables (PASC defined as U09.9 or Long-COVID Clinic Visit)

eTable 13. Comparison of Feature Importance Across Models for Not-Hospitalized during Index COVID for Unrestricted Sample including SDOH variables

eTable 14. Characteristics of Cohorts for U09.9 only

eTable 15. PASC Risk Factors from Logistic Regression (PASC defined as U09.9)

eTable 16. Characteristics of Cohorts for Long-COVID Clinic Visits

eTable 17. PASC Risk Factors from Logistic Regression (PASC defined as Long-COVID Clinic Visits)

eTable 18. Comparison of U09.9 and Long-COVID Clinic Visit Cohorts

**eMethods**

**Data**

We used the National COVID Cohort Collaborative (N3C) data in this study. It has been used extensively to study risk factors, medication use, and long-term health consequences of COVID-19 [20-23].

We used a limited version of N3C data. Data from numerical lab values were harmonized into canonical units of measure as previously described [20]. Site-level quality filters were applied prior to analysis in order to remove 13 of the 72 data partners with systematic missingness according to the following criteria: sites (1) should not shift dates by more than 30 days, (2) should have serum creatinine and lymphocyte count results for at least 25% of hospitalizations, and (3) should not have >10% of their hospitalizations where the COVID-19 index date is more than 200 days after the visit start date.

The N3C data transfer to NCATS is performed under Johns Hopkins University Reliance Protocol #IRB00249128 or individual site agreements with NIH. The N3C Data Enclave is managed under the authority of the NIH; information can be found at <https://ncats.nih.gov/n3c/resources>.

**Case and control selection**

For two control cohorts, we applied our previously developed computable phenotype (CP) model for long-COVID to refine our control patient pool [23]. Our group has previously developed two distinct CPs: one for U09.9 diagnosis and another for long-COVID clinic visits. Both CP models have moderate discrimination in classifying long-COVID (correctly categorizing patients as long-COVID cases versus controls based on patient symptoms several months after their infection) and are well-calibrated. Briefly, CP model did not use any data from 45 days before and 45 days after COVID-19 diagnosis date (day 0). It only counted diagnoses that newly occurred or occurred in greater frequency in the post-COVID-19 period (days 91 through 365) compared with the pre-COVID-19 period (days -91 through -365), and only counted medications that were newly prescribed in the post-COVID-19 period, with no order records in the pre-COVID-19 period. We defined greater frequency of diagnosis if someone had more instances of a particular diagnosis codes in the post-COVID-19 period compared to pre-COVID-19 period. We retrained these CP models by excluding risk factors we were interested in evaluating in this study. We applied CP model to the 1,054,336 non-cases (1,062,661 - 8,325) to generate a predicted probability for U09.9 diagnosis or long-COVID clinic visit. Importantly, to leverage the CP models for refining our control cases while minimizing circularity, we retrained that model without the 4 pre-existing chronic conditions it had previously used (end-stage renal disease, obstructive sleep apnea, multiple sclerosis, and diabetes mellitus types 1 and 2).

In each of the three matching methods, we randomly matched 1 case to 5 controls from the same health system without replacement. To achieve temporal alignment during different pandemic phases, we also matched on COVID index date within +/- 45 days of the corresponding case's earliest COVID index date. In the “unrestricted” method, we matched 8,325 cases to 41,625 controls. In the “restricted” and “more restricted controls” methods, we matched 8,322 cases to 41,610 controls; we lost 3 cases because they did not match 5 controls.

**Risk factors**

To identify comorbidities in N3C, we created concept sets using the primary conditions listed in the Systematized Nomenclature of Medicine – Clinical Terms (SNOMED-CT) hierarchy and included all descendants of the key related concepts (concept sets are reproducible in the N3C Enclave and can be surfaced externally in GitHub). Because this analysis is focused on risk factors, we only included reported instances of these comorbidities that were recorded any time prior to or on the COVID-19 index date.

It is challenging to determine if an individual was hospitalized for or with SARS-CoV-2 infection. To address this, we applied the following criteria to flag an inpatient stay as being associated with COVID-19: the patient was hospitalized +/-14 days from their (a) first positive SARS-CoV-2 PCR or AG lab test OR (b) the first COVID-19 diagnosis (U07.1) that was charted during an inpatient stay or emergency room visit. For individuals hospitalized within +/- 14 days of their index date for COVID-19 infection, we used information from the acute hospitalized phase, i.e, between COVID-19 hospitalization admission and discharge date, and identified invasive mechanical ventilation (IMV) use, extracorporeal membrane oxygenation (ECMO) use, vasopressor use, acute kidney injury diagnosis, sepsis diagnosis, remdesivir use, and total length of hospital stay. The set of comorbidities recorded up to and at the time of infection, the events during the COVID-19-associated hospitalization, and basic patient demographics were included as risk factors in all analyses.

For SDoH, we used county-level variables from the Sharecare-Boston University School of Public Health Social Determinants of Health dataset [26] linked to patients based on the preferred county (majority residence) associated with the patient’s 5-digit ZIP code. Because joining these data requires a five digit zip code, these fields are only available for patients with a five digit zip code. This dataset provides measures across domains identified by the CDC Healthy People 2020 framework: economic stability, education, health and healthcare. We operationalized these variables as: percent of households with income below poverty, percent of residents with college degree, percent of residents 19-64 with public insurance, and physicians per 1000 residents [26]. These are all included as tertiles in the analyses.

**Statistical analysis**

In addition to logistic regression, we used two machine learning methods, random forest (RF) [27] and XGBoost, to identify influential risk factors for developing PASC [28]. Machine learning methods provide the ability to investigate massive datasets and reveal patterns within data without relying on a priori assumptions such as pre-specified statistical interactions, specific variable associations, or linearity in variable relationships [29]. Each machine learning model used 5-fold cross-validation (K = 5) repeated 5 times to decrease overfitting. We used the average area under the receiver operator curve (AUC) as the primary metric to evaluate model performance. Model performance was important to establish as it relates to the effectiveness of the models in their ranking of risk factors associated with the outcome (PASC). We also conducted feature importance analysis for both RF and XGBoost models [30]. See the Supplement for hyperparameter tuning information. We calculated the average of the feature importance scores across the folds to determine which PASC risk factors were most influential. We also display feature importance and estimated SHAP (SHapley Additive exPlanations) plots [31] from the XGboost models (SHAP plots not shown, underlying data shown in Table 2).

For the unrestricted controls and PASC cases defined by U09.9 or a long-COVID visit (primary cohort), we stratified LR, XGBoost, and RF models by patients who were and were not hospitalized at the time of acute COVID infection to assess whether risk factors differed for these two groups.

Hyperparameter Tuning of Machine Learning Models: As XGBoost and random forest models consist of many decision trees, we fine-tuned the tree-specific hyperparameters with ensemble hyperparameters. After multiple iterations, we found our best performing XGBoost classifier model with the following hyperparameters: colsample_bytree=0.1, gamma=0.4, learning_rate=0.09, max_depth=8, min_child_weight=0, n_estimators=400, subsample=0.9. For the random forest classifier, the best performing model had the following hyperparameters: n_estimators=32, verbose=1, criterion='entropy’. In both cases random_state was set to 42.

**Secondary and stratified analysis**

In the primary cohort (PASC cases defined by U09.9 and a long-COVID visit and unrestricted controls), we then included SDoH variables and refit each of the three model types. These comparisons were important because SDoH have been associated with racial/ethnic disparities in COVID incidence, COVID severity, and development of PASC [26, 32].

**eResults**

**Sensitivity Analysis: Other Definitions of PASC**

Cases defined as U09.9 When we conducted a sensitivity analysis using only the 7,512 cases identified using ICD10 code U09.9, the magnitudes of PASC risk factors were largely similar to those observed in the primary analysis (eTable 14 and eTable 15). For example, patients between 40 and 69 years, female patients, and patients with comorbidities such as chronic lung disease, rheumatologic disease, and peptic ulcer had higher likelihood of PASC diagnosis. Risk factors negatively associated with PASC include Non-Hispanic Black race and Hispanic ethnicity and behavioral risk factors. As before, the XGBoost indicated similar feature importance and direction of the associations (eFigure 1 Panel B).

Cases defined by long-COVID clinic Visit We then conducted a sensitivity analysis using only data from the 5 health systems that reported long-COVID clinic visits. We matched 1,241 cases to 6,205 controls. We found similar results in this analysis (eTable 16 and eTable 17). However, confidence intervals for some risk factors were wide due to small sample size and limited power. XGBoost also showed similar features with this outcome (eFigure 1 Panel C).

Comparison of Risk Factors across PASC definitions In eTable 18 we compare the three case definitions of PASC (U09.9 alone, long-COVID visit alone, or either). Risk factors across the three possible definitions are fairly similar (eTable 2, eTable 18, and eTable 15). Across logistic regression models, statistical precision varies in part by sample size. The U09.9 sample appears to dominate the long-COVID visit sample in the combined model.

**eFigures**

**eFigure1. Feature Importance XGBoost (PASC defined as U09.9 or Long-COVID Clinic Visit)**

**Panel A Unrestricted Controls (Method 1)**

**
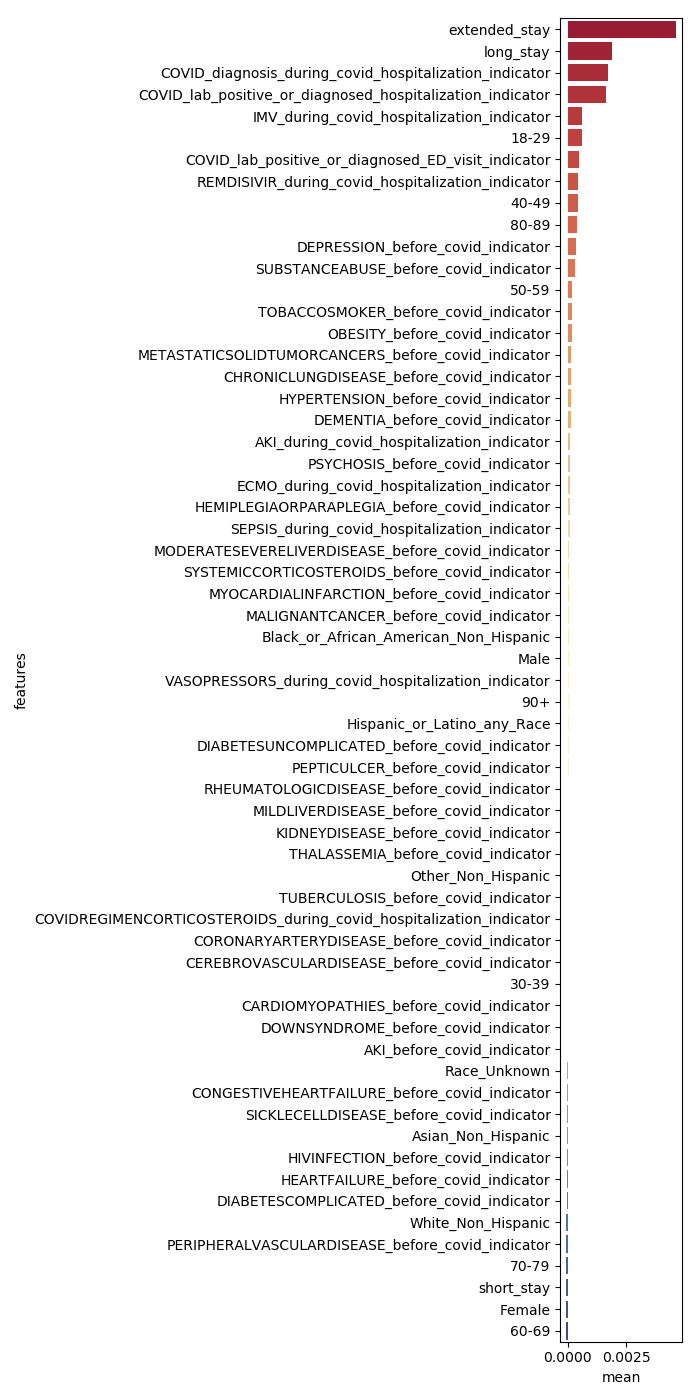
**

**Panel B Less Restricted Controls (Method 2)**

**
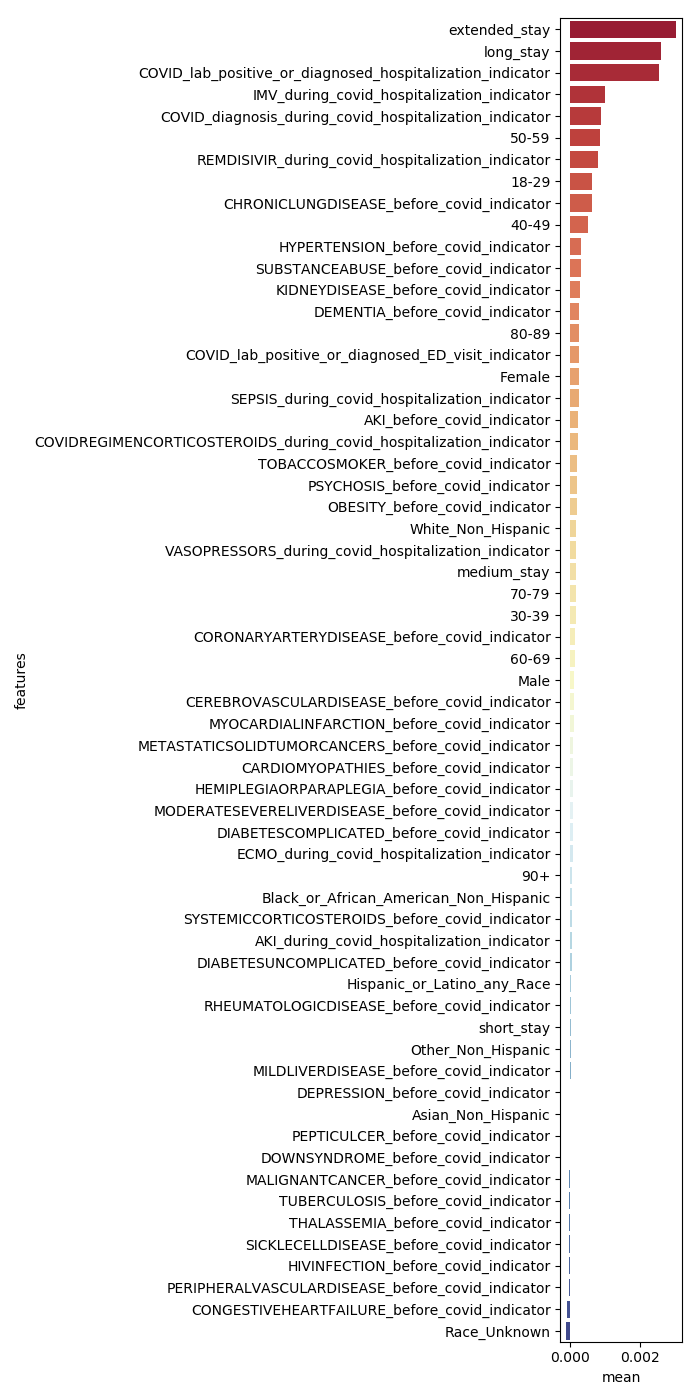
**

**Panel C More Restricted Controls (Method 3)**

**
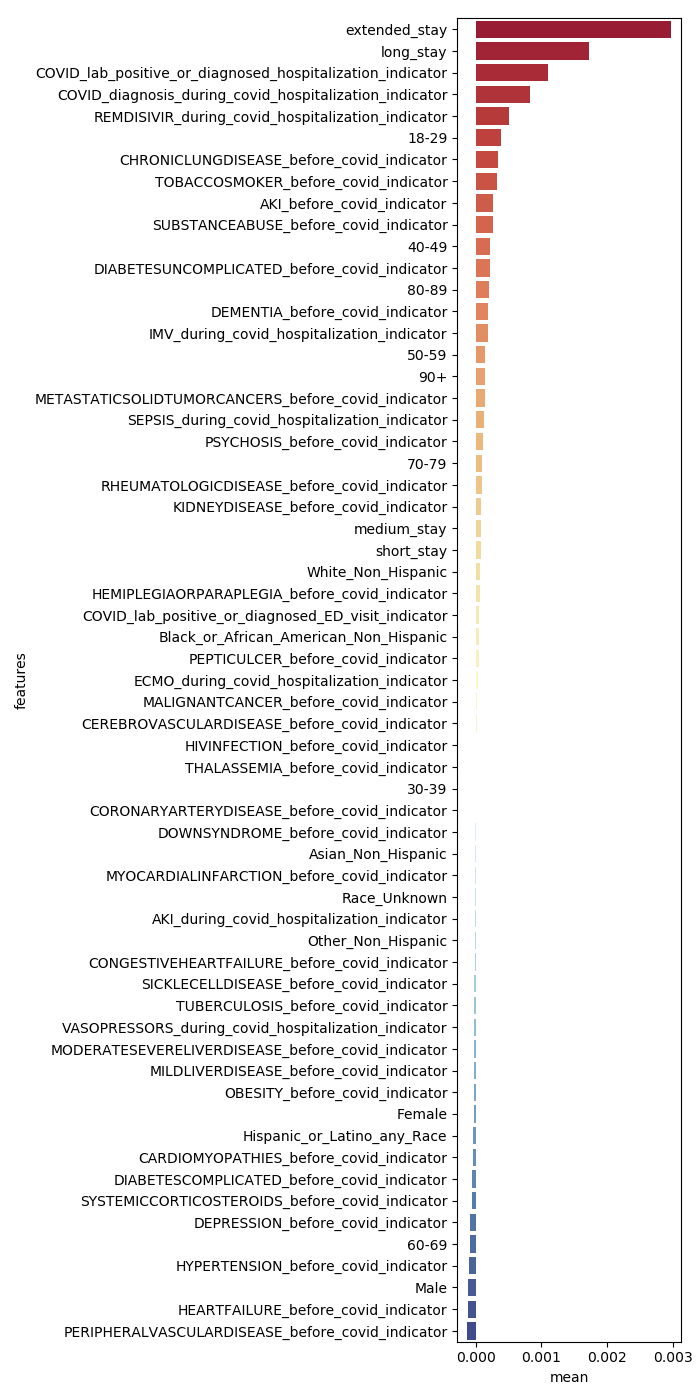
**

**eFigure 2. Forest Plot for Unrestricted Controls without SDoH (PASC defined as U09.9 or Long-COVID Clinic Visit)**

**
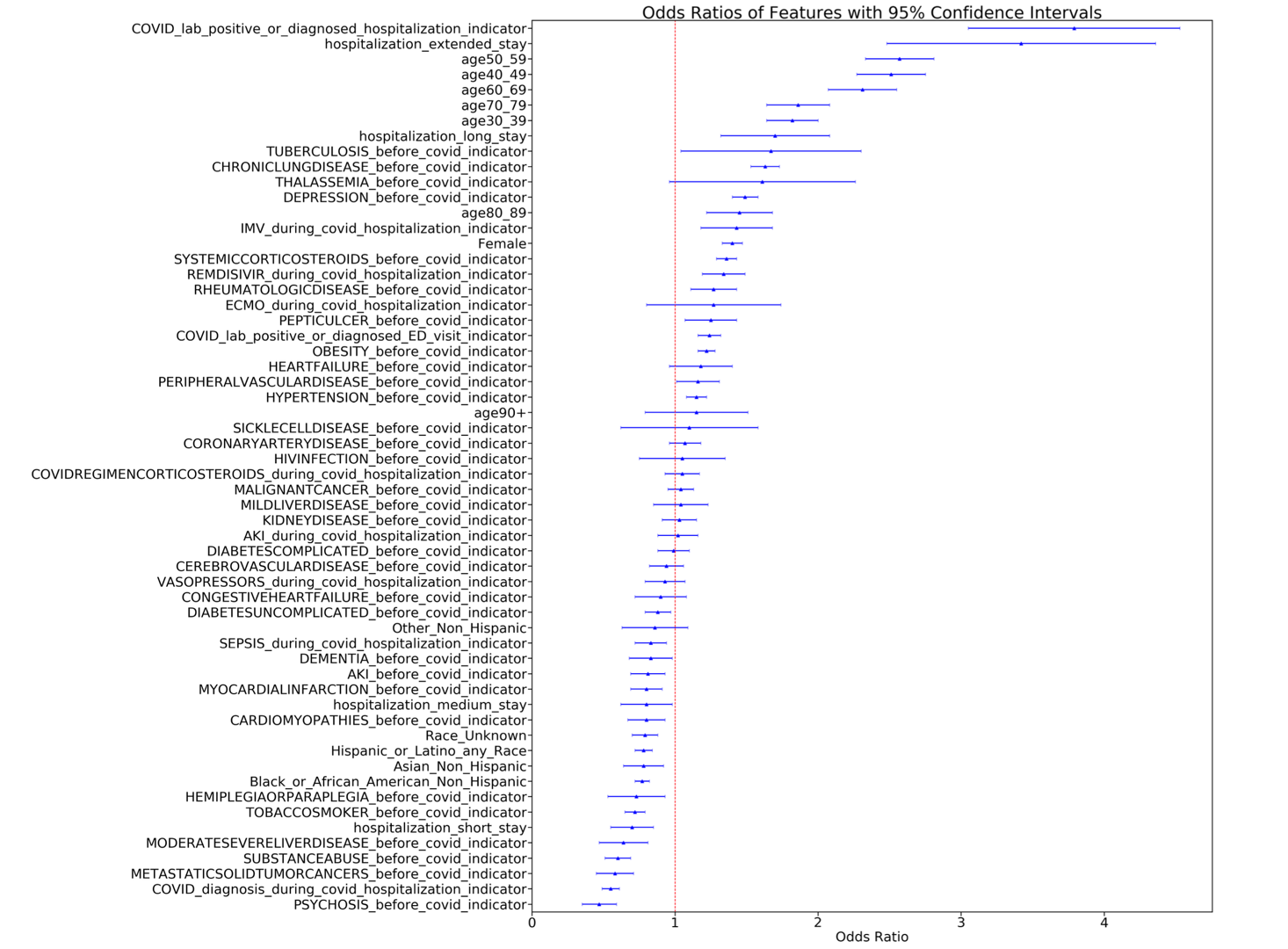
**

## **eFigure 3. Forest Plot for Age association (Male)**

##
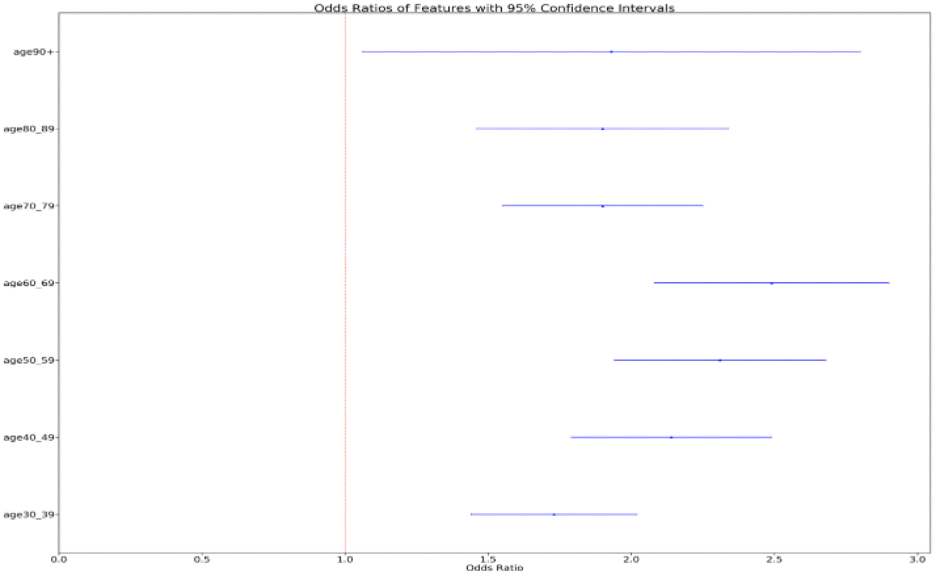


## **eFigure 4. Forest Plot for Age association (Female)**

**
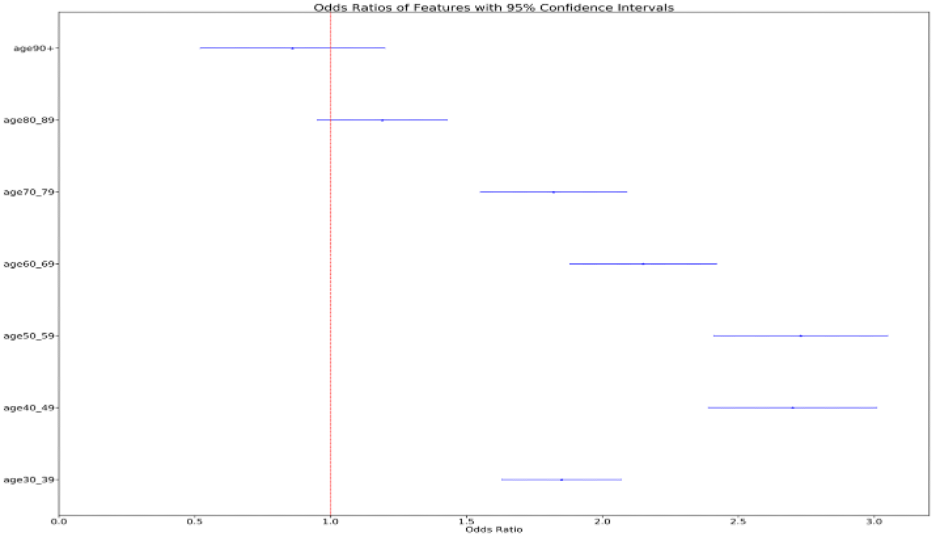
**

**eFigure 5. Feature Importance XGBoost for Unrestricted Controls with SDoH (PASC defined as U09.9 or Long-COVID Clinic Visit)**

**
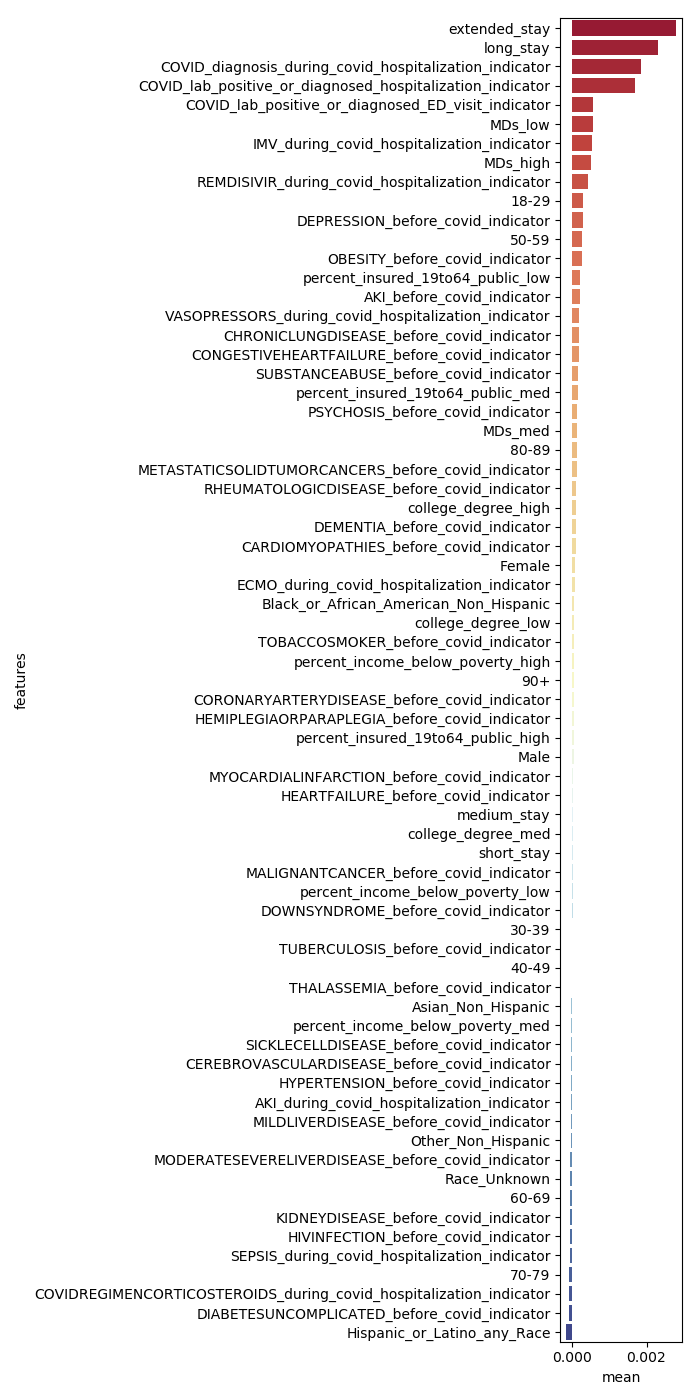
**

**eFigure 6. Feature Importance XGBoost for Unrestricted Controls with SDoH–Hospitalized Sample (PASC defined as U09.9 or Long COVID Visit)**

**
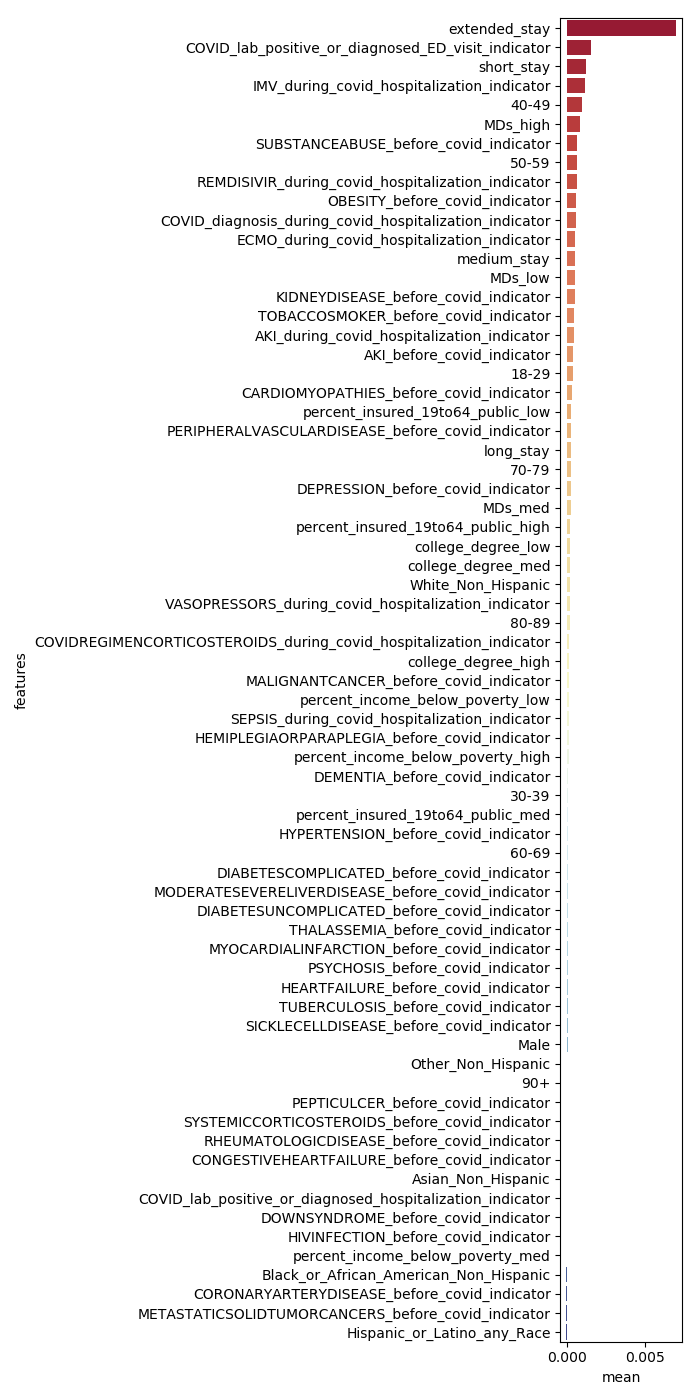
**

**eFigure 7. Feature Importance XGBoost for Unrestricted Controls with SDoH –non-Hospitalized Sample (PASC defined as U09.9 or Long COVID Visit)**

**
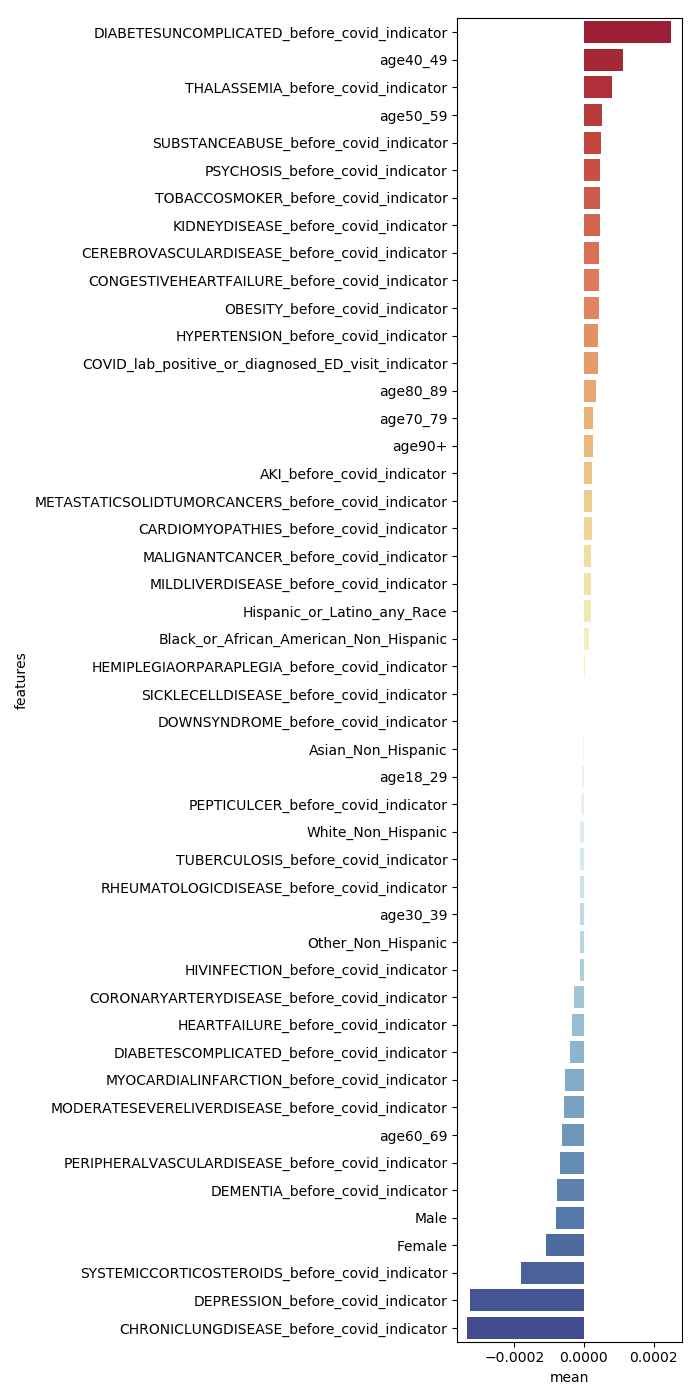
**

## **eTables**

**eTable 1. Cohort Characteristics for PASC cases defined by U09.9 or clinic visit (Additional Characteristics Not Shown in Table 1)**

|  | PASC  (N=8325) | Method 1  Unrestricted controls (N=41625) | Method 2  Restricted controls (N=41610) | Method 3  Most restricted controls (N=41610) |
| --- | --- | --- | --- | --- |
| **Demographics** |  |  |  |  |
| Age |  |  |  |  |
| 18-29 | 630 (7.57%) | 8366 (20.1%) | 8351 (20.1%) | 7887 (19.0%) |
| 30-39 | 1229 (14.8%) | 7920 (19.0%) | 7823 (18.8%) | 7426 (17.8%) |
| 40-49 | 1749 (21.0%) | 7321 (17.6%) | 6808 (16.4%) | 7022 (16.9%) |
| 50-59 | 1933 (23.2%) | 7171 (17.2%) | 6842 (16.4%) | 7161 (17.2%) |
| 60-69 | 1605 (19.3%) | 5806 (13.9%) | 5990 (14.4%) | 6400 (15.4%) |
| 70-79 | 840 (10.1%) | 3377 (8.1%) | 3773 (9.1%) | 3742 (9.0%) |
| 80-89 | 293 (3.5%) | 1391 (3.3%) | 1673 (4.0%) | 1588 (3.8%) |
| 90+ | 37 (0.4%) | 224 (0.5%) | 287 (0.7%) | 305 (0.7%) |
| **Comorbidities Prior to COVID Index Date** |  |  |  |  |
| AKI | 862 (10.4%) | 2287 (5.5%) | 2783 (6.7%) | 2738 (6.6%) |
| Cardiomyopathies | 225 (2.7%) | 768 (1.8%) | 927 (2.2%) | 897 (2.2%) |
| Cerebrovascular Disease | 390 (4.7%) | 1297 (3.1%) | 1707 (4.1%) | 1647 (4.0%) |
| Coronary Artery Disease | 832 (10.0%) | 2569 (6.2%) | 3319 (8.0%) | 3307 (7.9%) |
| Dementia | 153 (1.8%) | 643 (1.5%) | 846 (2.0%) | 832 (2.0%) |
| Down Syndrome | <20 | <20 | <20 | <20 |
| Heart Failure | 737 (8.9%) | 1936 (4.7%) | 2473 (5.9%) | 2375 (5.7%) |
| Hemiplegia or Paraplegia | 61 (0.7%) | 240 (0.6%) | 313 (0.8%) | 344 (0.8%) |
| HIV | 51 (0.6%) | 214 (0.5%) | 307 (0.7%) | 341 (0.8%) |
| Malignant Cancer | 837 (10.1%) | 2879 (6.9%) | 3826 (9.2%) | 3772 (9.1%) |
| Metastatic Solid Tumor Cancers | 91 (1.1%) | 426 (1.0%) | 568 (1.4%) | 551 (1.3%) |
| Mild Liver Disease | 170 (2.0%) | 600 (1.4%) | 780 (1.9%) | 791 (1.9%) |
| Moderate to Severe Liver Disease | 82 (1.0%) | 305 (0.7%) | 391 (0.9%) | 353 (0.8%) |
| Myocardial Infarction | 392 (4.7%) | 1311 (3.1%) | 1616 (3.9%) | 1659 (4.0%) |
| Peptic Ulcer | 279 (3.4%) | 714 (1.7%) | 915 (2.2%) | 954 (2.3%) |
| Peripheral Vascular Disease | 405 (4.9%) | 1045 (2.5%) | 1453 (3.5%) | 1362 (3.3%) |
| Rheumatologic Disease | 350 (4.2%) | 804 (1.9%) | 1020 (2.5%) | 981 (2.4%) |
| Sickle Cell Disease | <20 | 64 (0.2%) | 95 (0.2%) | 77 (0.2%) |
| Systemic Corticosteroids | 4325 (52.0%) | 13754 (33.0%) | 16385 (39.4%) | 16177 (38.9%) |
| Thalassemia | 21 (0.3%) | 66 (0.2%) | 78 (0.2%) | 89 (0.2%) |
| Tuberculosis | 27 (0.3%) | 69 (0.2%) | 107 (0.3%) | 90 (0.2%) |
| **Behavioral Health Indicators** |  |  |  |  |
| Depression | 2059 (24.7%) | 5851 (14.1%) | 7649 (18.4%) | 7526 (18.1%) |
| Psychosis | 65 (0.8%) | 416 (1.0%) | 483 (1.2%) | 471 (1.1%) |
| Substance Abuse | 205 (2.5%) | 1291 (3.1%) | 1541 (3.7%) | 1526 (3.7%) |
| Tobacco Smoker | 515 (6.2%) | 2656 (6.4%) | 3065 (7.4%) | 3122 (7.5%) |
| **Characteristics during Acute COVID Phase** |  |  |  |  |
| COVID Diagnosis during COVID-associated Hospitalization | 2065 (24.8%) | 4668 (11.2%) | 4534 (10.9%) | 4448 (10.7%) |
| Hospitalization stay |  |  |  |  |
| Short Stay (0-2 days) | 610 (7.3%) | 2155 (5.2%) | 2158 (5.2%) | 2188 (5.3%) |
| Medium Stay (3-7 days) | 870 (10.5%) | 2226 (5.3%) | 2282 (5.5%) | 2141 (5.1%) |
| Long Stay (8-30 days) | 1029 (12.4%) | 1274 (3.1%) | 1309 (3.1%) | 1270 (3.1%) |
| Extended Stay (31+ days) | 449 (5.4%) | 241 (0.6%) | 239 (0.6%) | 216 (0.5%) |

^a^Only captured for individuals hospitalized for COVID-19

**eTable 2. PASC Risk Factors from Logistic Regression (PASC defined as U09.9 or long-COVID Clinic Visit)**

|  | Method 1  Unrestricted Controls  (N=49950) | Method 2  Restricted  controls  (N=49932) | Method 3  Most Restricted  controls  (N=49932) |
| --- | --- | --- | --- |
| **Demographics** |  |  |  |
| Age |  |  |  |
| 18-29 | REF | REF | REF |
| 30-39 | 1.82 (1.64-2.02) | 1.96 (1.77-2.18) | 1.99 (1.79-2.21) |
| 40-49 | 2.51 (2.27-2.77) | 3.08 (2.78-3.4) | 2.81 (2.54-3.11) |
| 50-59 | 2.57 (2.33-2.85) | 3.25 (2.94-3.59) | 2.9 (2.62-3.21) |
| 60-69 | 2.31 (2.07-2.57) | 2.74 (2.46-3.05) | 2.47 (2.22-2.75) |
| 70-79 | 1.86 (1.64-2.11) | 2.15 (1.9-2.44) | 2.0 (1.76-2.26) |
| 80-89 | 1.45 (1.22-1.72) | 1.57 (1.33-1.85) | 1.56 (1.32-1.85) |
| 90+ | 1.15 (0.79-1.69) | 1.06 (0.73-1.53) | 0.93 (0.64-1.35) |
| Sex |  |  |  |
| Male or Unknown Sex | REF | REF | REF |
| Female | 1.4 (1.33-1.48) | 1.33 (1.26-1.41) | 1.28 (1.21-1.35) |
| Race/ethnicity |  |  |  |
| White NH | REF | REF | REF |
| Hispanic | 0.78 (0.72-0.85) | 0.86 (0.79-0.93) | 0.83 (0.76-0.9) |
| Black NH | 0.77 (0.72-0.83) | 0.81 (0.75-0.87) | 0.78 (0.72-0.84) |
| Asian NH | 0.78 (0.64-0.95) | 0.72 (0.59-0.87) | 0.73 (0.6-0.88) |
| Other race NH | 0.86 (0.63-1.17) | 0.99 (0.72-1.36) | 0.98 (0.72-1.34) |
| Unknown | 0.79 (0.7-0.89) | 1.12 (0.98-1.27) | 1.09 (0.96-1.24) |
| **Comorbidities Prior to COVID Index Date** |  |  |  |
| AKI | 0.81 (0.69-0.95) | 0.84 (0.72-0.98) | 0.8 (0.68-0.93) |
| Cardiomyopathies | 0.8 (0.67-0.96) | 0.92 (0.77-1.1) | 0.88 (0.73-1.05) |
| Cerebrovascular Disease | 0.94 (0.82-1.08) | 0.87 (0.76-0.99) | 0.91 (0.8-1.04) |
| Chronic Lung Disease | 1.63 (1.53-1.74) | 1.57 (1.47-1.67) | 1.63 (1.53-1.74) |
| Complicated Diabetes | 0.99 (0.88-1.12) | 0.99 (0.87-1.11) | 1.07 (0.94-1.21) |
| Congestive Heart Failure | 0.9 (0.72-1.13) | 0.87 (0.69-1.09) | 0.9 (0.72-1.13) |
| Coronary Artery Disease | 1.07 (0.96-1.2) | 1.03 (0.93-1.15) | 1.03 (0.92-1.14) |
| Dementia | 0.83 (0.68-1.02) | 0.74 (0.61-0.9) | 0.76 (0.63-0.92) |
| Down Syndrome | 3.97 (0.53-30.01) | 1.74 (0.41-7.43) | 3.1 (0.6-16.01) |
| Heart Failure | 1.18 (0.96-1.45) | 1.18 (0.96-1.45) | 1.19 (0.97-1.46) |
| Hemiplegia or Paraplegia | 0.73 (0.53-0.99) | 0.7 (0.51-0.95) | 0.53 (0.39-0.72) |
| HIV | 1.05 (0.75-1.46) | 0.85 (0.62-1.17) | 0.77 (0.57-1.06) |
| Hypertension | 1.15 (1.08-1.23) | 0.98 (0.92-1.05) | 0.98 (0.92-1.05) |
| Kidney Disease | 1.03 (0.91-1.17) | 1.03 (0.91-1.16) | 1.07 (0.95-1.21) |
| Malignant Cancer | 1.04 (0.95-1.14) | 0.93 (0.85-1.02) | 0.94 (0.85-1.03) |
| Metastatic Solid Tumor Cancers | 0.58 (0.45-0.76) | 0.61 (0.47-0.78) | 0.6 (0.47-0.77) |
| Mild Liver Disease | 1.04 (0.85-1.28) | 0.93 (0.76-1.14) | 0.88 (0.72-1.07) |
| Moderate to Severe Liver Disease | 0.64 (0.47-0.85) | 0.64 (0.48-0.85) | 0.76 (0.57-1.03) |
| Myocardial Infarction | 0.8 (0.69-0.93) | 0.79 (0.68-0.91) | 0.77 (0.66-0.88) |
| Obesity | 1.22 (1.16-1.29) | 1.05 (0.99-1.1) | 1.06 (1.0-1.12) |
| Peptic Ulcer | 1.25 (1.07-1.45) | 1.23 (1.06-1.42) | 1.17 (1.01-1.35) |
| Peripheral Vascular Disease | 1.16 (1.0-1.34) | 1.06 (0.92-1.21) | 1.11 (0.97-1.27) |
| Rheumatologic Disease | 1.27 (1.11-1.46) | 1.22 (1.07-1.4) | 1.31 (1.14-1.5) |
| Sickle Cell Disease | 1.1 (0.62-1.97) | 0.87 (0.5-1.51) | 0.89 (0.5-1.57) |
| Systemic Corticosteroids | 1.36 (1.29-1.44) | 1.19 (1.13-1.26) | 1.21 (1.15-1.28) |
| Thalassemia | 1.61 (0.96-2.7) | 1.4 (0.83-2.35) | 1.2 (0.72-2.0) |
| Tuberculosis | 1.67 (1.04-2.67) | 1.15 (0.73-1.8) | 1.53 (0.97-2.41) |
| Uncomplicated Diabetes | 0.88 (0.79-0.98) | 0.87 (0.79-0.97) | 0.82 (0.74-0.91) |
| **Behavioral Health** |  |  |  |
| Depression | 1.49 (1.4-1.59) | 1.31 (1.23-1.39) | 1.34 (1.26-1.43) |
| Psychosis | 0.47 (0.35-0.62) | 0.56 (0.42-0.74) | 0.56 (0.42-0.74) |
| Substance Abuse | 0.6 (0.51-0.71) | 0.59 (0.5-0.69) | 0.59 (0.5-0.7) |
| Tobacco Smoker | 0.72 (0.65-0.8) | 0.72 (0.65-0.8) | 0.66 (0.6-0.74) |
| **Characteristics of Index COVID "Acute Phase"** |  |  |  |
| COVID Diagnosis during COVID-associated Hospitalization | 0.55 (0.49-0.62) | 0.73 (0.66-0.81) | 0.68 (0.61-0.76) |
| COVID-associated Hospitalization | 3.79 (3.05-4.72) | 3.35 (2.72-4.14) | 3.08 (2.5-3.79) |
| COVID-associated ED Visit | 1.24 (1.16-1.33) | 1.4 (1.31-1.5) | 1.44 (1.34-1.54) |
| Hospitalization stay |  |  |  |
| Not Hospitalized | REF | REF | REF |
| Short Stay | 0.7 (0.55-0.88) | 0.67 (0.53-0.85) | 0.75 (0.6-0.94) |
| Medium Stay | 0.8 (0.62-1.02) | 0.74 (0.58-0.94) | 0.87 (0.69-1.09) |
| Long Stay | 1.7 (1.32-2.18) | 1.54 (1.2-1.96) | 1.78 (1.4-2.27) |
| Extended Stay | 3.42 (2.48-4.71) | 2.76 (2.01-3.79) | 3.43 (2.5-4.71) |
| COVID treatment |  |  |  |
| Corticosteroidsᵃ | 1.05 (0.93-1.18) | 1.14 (1.01-1.28) | 1.2 (1.07-1.35) |
| Remdesivirᵃ | 1.34 (1.19-1.51) | 1.53 (1.36-1.72) | 1.59 (1.41-1.79) |
| Vasopressorsᵃ | 0.93 (0.79-1.1) | 0.9 (0.77-1.06) | 0.81 (0.69-0.95) |
| ECMOᵃ | 1.27 (0.8-2.01) | 1.67 (1.0-2.79) | 2.59 (1.46-4.62) |
| Mechanical Ventilationᵃ | 1.43 (1.18-1.74) | 1.73 (1.42-2.09) | 1.6 (1.32-1.94) |
| AKI during COVID-associated Hospitalization | 1.02 (0.88-1.19) | 1.0 (0.86-1.16) | 1.03 (0.88-1.19) |
| Sepsis during COVID-associated Hospitalization | 0.83 (0.72-0.96) | 0.81 (0.7-0.93) | 0.86 (0.75-1.0) |

ᵃOnly captured for individuals hospitalized for COVID-19

Odds ratios presented with 95% CI in parenthesis

**eTable 3. AUC scores for our three models of PASC defined by U09.9 or Long-COVID Clinic Visit**

| **Cohorts** | **XGBoost (AUC score)** | **RF (AUC score)** | **LR (AUC score)** |
| --- | --- | --- | --- |
| Cohort A | 0.714 | 0.659 | 0.709 |
| Cohort B | 0.731 | 0.678 | 0.726 |
| Cohort C | 0.712 | 0.659 | 0.708 |

**eTable 4. Comparison of Feature Importance Across Models for Unrestricted Sample (PASC defined as U09.9 or Long-COVID Clinic Visit)**

| features | Logistic Regression | Random Forest | XGBoost | Mean Rank |
| --- | --- | --- | --- | --- |
| Hospitalization Extended Stay (31+ days) | 2 | 4 | 1 | 2.33 |
| COVID-associated Hospitalization | 4 | 1 | 4 | 3.00 |
| COVID Diagnosis during COVID-associated Hospitalization | 10 | 5 | 3 | 6.00 |
| Age 40-49 | 6 | 7 | 9 | 7.33 |
| Age 50-59 | 5 | 6 | 13 | 8.00 |
| Hospitalization Long Stay (8-30 days) | 7 | 19 | 2 | 9.33 |
| Age 18-29 | ref. | 13 | 6 | 9.50 |
| Chronic Lung Disease | 16 | 9 | 18 | 14.33 |
| Age 60-69 | 8 | 11 | 27 | 15.33 |
| Depression | 19 | 16 | 11 | 15.33 |
| Female | 22 | 3 | 29 | 18.00 |
| COVID Treatment: Mechanical Ventilation | 21 | 29 | 5 | 18.33 |
| COVID Treatment: Remdesivir | 26 | 22 | 8 | 18.67 |
| Male | ref. | 2 | 36 | 19.00 |
| Age 70-79 | 11 | 20 | 31 | 20.67 |
| Substance Abuse | 15 | 39 | 12 | 22.00 |
| COVID-associated ED Visit | 37 | 23 | 7 | 22.33 |
| Race/Ethnicity: White NH | ref. | 10 | 37 | 23.50 |
| Race/Ethnicity: Black NH | 27 | 12 | 34 | 24.33 |
| Metastatic Solid Tumor Cancers | 13 | 46 | 17 | 25.33 |
| Psychosis | 9 | 45 | 22 | 25.33 |
| Tobacco Smoker | 23 | 38 | 15 | 25.33 |
| Age 30-39 | 12 | 8 | 59 | 26.33 |
| Systemic Corticosteroids | 25 | 30 | 28 | 27.67 |
| Age 80-89 | 20 | 56 | 10 | 28.67 |
| Myocardial Infarction | 33 | 21 | 32 | 28.67 |
| Dementia | 40 | 26 | 20 | 28.67 |
| Moderate to Severe Liver Disease | 18 | 50 | 26 | 31.33 |
| Hospitalization Short Stay (0-2 days) | ref. | 34 | 30 | 32.00 |
| Race/Ethnicity: Hispanic | 29 | 25 | 42 | 32.00 |
| Hemiplegia or Paraplegia | 24 | 52 | 24 | 33.33 |
| Obesity | 38 | 47 | 16 | 33.67 |
| Malignant Cancer | 55 | 14 | 33 | 34.00 |
| Uncomplicated Diabetes | 46 | 15 | 44 | 35.00 |
| Hypertension | 45 | 43 | 19 | 35.67 |
| Race/Ethnicity: Asian NH | 28 | 33 | 46 | 35.67 |
| Rheumatologic Disease | 30 | 31 | 47 | 36.00 |
| COVID Treatment: ECMO | 31 | 54 | 23 | 36.00 |
| Cardiomyopathies | 34 | 18 | 60 | 37.33 |
| Peripheral Vascular Disease | 43 | 36 | 35 | 38.00 |
| Hospitalization Medium Stay (3-7 days) | 49 | 51 | 14 | 38.00 |
| Sepsis during COVID-associated Hospitalization | 39 | 57 | 25 | 40.33 |
| Race/Ethnicity: Unknown | 32 | 32 | 57 | 40.33 |
| Coronary Artery Disease | 50 | 17 | 56 | 41.00 |
| Heart Failure | 41 | 42 | 40 | 41.00 |
| Thalassemia | 17 | 58 | 50 | 41.67 |
| Complicated Diabetes | 59 | 28 | 38 | 41.67 |
| Tuberculosis | 14 | 59 | 54 | 42.33 |
| Down Syndrome | 3 | 62 | 62 | 42.33 |
| AKI during COVID-associated Hospitalization | 58 | 49 | 21 | 42.67 |
| Kidney Disease | 57 | 24 | 49 | 43.33 |
| COVID Treatment: Vasopressors | 51 | 44 | 39 | 44.67 |
| Mild Liver Disease | 54 | 35 | 48 | 45.67 |
| COVID Treatment: Corticosteroids | 56 | 27 | 55 | 46.00 |
| Age 90+ | 44 | 53 | 41 | 46.00 |
| AKI prior to COVID | 36 | 41 | 61 | 46.00 |
| Congestive Heart Failure | 47 | 40 | 52 | 46.33 |
| Peptic Ulcer | 35 | 60 | 45 | 46.67 |
| Race/Ethnicity: Other race NH | 42 | 48 | 53 | 47.67 |
| Cerebrovascular Disease | 52 | 37 | 58 | 49.00 |
| HIV | 53 | 55 | 43 | 50.33 |
| Sickle Cell Disease | 48 | 61 | 51 | 53.33 |

**eTable 5. Comparison of Feature Importance Across Models for Restricted Sample (PASC defined as U09.9 or Long-COVID Clinic Visit)**

| features | Logistic Regression | Random Forest | XGBoost | Mean Rank |
| --- | --- | --- | --- | --- |
| COVID-associated Hospitalization | 6 | 1 | 3 | 3.33 |
| Hospitalization Extended Stay (31+ days) | 2 | 9 | 1 | 4.00 |
| Age 50-59 | 3 | 5 | 6 | 4.67 |
| Age 18-29 | ref. | 11 | 8 | 6.33 |
| Age 40-49 | 4 | 6 | 10 | 6.67 |
| Hospitalization Long Stay (8-30 days) | 7 | 18 | 2 | 9.00 |
| COVID Treatment: Mechanical Ventilation | 12 | 15 | 4 | 10.33 |
| Chronic Lung Disease | 16 | 7 | 9 | 10.67 |
| Male | ref. | 3 | 31 | 11.33 |
| Race/Ethnicity: White NH | ref. | 13 | 24 | 12.33 |
| Age 60-69 | 5 | 4 | 30 | 13.00 |
| COVID Diagnosis during COVID-associated Hospitalization | 21 | 14 | 5 | 13.33 |
| Age 30-39 | 9 | 8 | 28 | 15.00 |
| Female | 27 | 2 | 17 | 15.33 |
| COVID Treatment: Remdesivir | 19 | 24 | 7 | 16.67 |
| Age 70-79 | 8 | 16 | 27 | 17.00 |
| COVID-associated ED Visit | 25 | 17 | 16 | 19.33 |
| Substance Abuse | 13 | 38 | 12 | 21.00 |
| Tobacco Smoker | 23 | 23 | 21 | 22.33 |
| Dementia | 26 | 29 | 14 | 23.00 |
| Psychosis | 10 | 37 | 22 | 23.00 |
| Hospitalization Short Stay (0-2 days) | ref. | 22 | 50 | 24.00 |
| Age 80-89 | 17 | 46 | 15 | 26.00 |
| Race/Ethnicity: Black NH | 31 | 10 | 43 | 28.00 |
| COVID Treatment: Corticosteroids | 44 | 25 | 20 | 29.67 |
| Sepsis during COVID-associated Hospitalization | 30 | 43 | 18 | 30.33 |
| Coronary Artery Disease | 54 | 12 | 29 | 31.67 |
| AKI prior to COVID | 34 | 45 | 19 | 32.67 |
| Metastatic Solid Tumor Cancers | 15 | 49 | 36 | 33.33 |
| Systemic Corticosteroids | 35 | 20 | 45 | 33.33 |
| Hypertension | 56 | 34 | 11 | 33.67 |
| Kidney Disease | 55 | 33 | 13 | 33.67 |
| Cardiomyopathies | 47 | 19 | 37 | 34.33 |
| Myocardial Infarction | 29 | 41 | 33 | 34.33 |
| Obesity | 53 | 27 | 23 | 34.33 |
| Cerebrovascular Disease | 40 | 32 | 32 | 34.67 |
| Race/Ethnicity: Unknown | 45 | 28 | 35 | 36.00 |
| Congestive Heart Failure | 39 | 26 | 44 | 36.33 |
| COVID Treatment: ECMO | 14 | 58 | 41 | 37.67 |
| Moderate to Severe Liver Disease | 18 | 59 | 39 | 38.67 |
| Complicated Diabetes | 57 | 21 | 40 | 39.33 |
| Heart Failure | 36 | 48 | 34 | 39.33 |
| Hemiplegia or Paraplegia | 20 | 60 | 38 | 39.33 |
| Race/Ethnicity: Asian NH | 24 | 39 | 56 | 39.67 |
| Uncomplicated Diabetes | 43 | 31 | 47 | 40.33 |
| COVID Treatment: Vasopressors | 46 | 51 | 25 | 40.67 |
| Race/Ethnicity: Hispanic | 37 | 42 | 48 | 42.33 |
| Peptic Ulcer | 32 | 40 | 57 | 43.00 |
| Hospitalization Medium Stay (3-7 days) | 52 | 52 | 26 | 43.33 |
| Depression | 28 | 50 | 55 | 44.33 |
| AKI during COVID-associated Hospitalization | 59 | 30 | 46 | 45.00 |
| Down Syndrome | 11 | 62 | 62 | 45.00 |
| Mild Liver Disease | 48 | 36 | 52 | 45.33 |
| Peripheral Vascular Disease | 50 | 35 | 53 | 46.00 |
| Rheumatologic Disease | 33 | 56 | 49 | 46.00 |
| Thalassemia | 22 | 57 | 59 | 46.00 |
| Age 90+ | 51 | 47 | 42 | 46.67 |
| HIV | 38 | 55 | 54 | 49.00 |
| Sickle Cell Disease | 41 | 54 | 58 | 51.00 |
| Malignant Cancer | 49 | 44 | 61 | 51.33 |
| Race/Ethnicity: Other race NH | 58 | 53 | 51 | 54.00 |
| Tuberculosis | 42 | 61 | 60 | 54.33 |

**eTable 6. Comparison of Feature Importance Across Models for More Restricted Sample (PASC defined as U09.9 or Long-COVID Clinic Visit)**

| features | Logistic Regression | Random Forest | XGBoost | Mean Rank |
| --- | --- | --- | --- | --- |
| COVID-associated Hospitalization | 8 | 1 | 3 | 4.00 |
| Hospitalization Extended Stay (31+ days) | 2 | 10 | 1 | 4.33 |
| Age 18-29 | ref. | 15 | 6 | 7.00 |
| Age 40-49 | 5 | 4 | 12 | 7.00 |
| Male | ref. | 2 | 24 | 8.67 |
| Age 50-59 | 4 | 6 | 17 | 9.00 |
| Hospitalization Long Stay (8-30 days) | 9 | 17 | 2 | 9.33 |
| Chronic Lung Disease | 16 | 12 | 7 | 11.67 |
| COVID Diagnosis during COVID-associated Hospitalization | 22 | 14 | 4 | 13.33 |
| Age 60-69 | 7 | 7 | 28 | 14.00 |
| Race/Ethnicity: White NH | ref. | 9 | 33 | 14.00 |
| Age 70-79 | 10 | 13 | 26 | 16.33 |
| COVID Treatment: Mechanical Ventilation | 17 | 23 | 16 | 18.67 |
| COVID Treatment: Remdesivir | 18 | 33 | 5 | 18.67 |
| Substance Abuse | 14 | 34 | 11 | 19.67 |
| Age 80-89 | 19 | 31 | 14 | 21.33 |
| Dementia | 26 | 25 | 15 | 22.00 |
| Hospitalization Short Stay (0-2 days) | ref. | 39 | 31 | 23.33 |
| Uncomplicated Diabetes | 35 | 24 | 13 | 24.00 |
| COVID Treatment: Corticosteroids | 40 | 26 | 9 | 25.00 |
| Tobacco Smoker | 21 | 46 | 8 | 25.00 |
| Metastatic Solid Tumor Cancers | 15 | 42 | 19 | 25.33 |
| Age 30-39 | 11 | 5 | 61 | 25.67 |
| Female | 32 | 3 | 43 | 26.00 |
| Race/Ethnicity: Black NH | 31 | 11 | 37 | 26.33 |
| Psychosis | 13 | 56 | 22 | 30.33 |
| AKI prior to COVID | 33 | 49 | 10 | 30.67 |
| Rheumatologic Disease | 28 | 37 | 27 | 30.67 |
| Systemic Corticosteroids | 36 | 21 | 35 | 30.67 |
| COVID-associated ED Visit | 23 | 35 | 36 | 31.33 |
| Hemiplegia or Paraplegia | 12 | 48 | 34 | 31.33 |
| Race/Ethnicity: Hispanic | 37 | 19 | 41 | 32.33 |
| Hypertension | 58 | 16 | 25 | 33.00 |
| Myocardial Infarction | 29 | 18 | 56 | 34.33 |
| Sepsis during COVID-associated Hospitalization | 42 | 40 | 21 | 34.33 |
| Heart Failure | 38 | 43 | 23 | 34.67 |
| Hospitalization Medium Stay (3-7 days) | 47 | 30 | 30 | 35.67 |
| Race/Ethnicity: Asian NH | 24 | 27 | 57 | 36.00 |
| COVID Treatment: ECMO | 6 | 62 | 42 | 36.67 |
| Complicated Diabetes | 53 | 20 | 38 | 37.00 |
| COVID Treatment: Vasopressors | 34 | 28 | 49 | 37.00 |
| Cardiomyopathies | 44 | 29 | 40 | 37.67 |
| Peripheral Vascular Disease | 48 | 45 | 20 | 37.67 |
| Depression | 25 | 57 | 32 | 38.00 |
| Kidney Disease | 52 | 38 | 29 | 39.67 |
| Age 90+ | 51 | 52 | 18 | 40.33 |
| Down Syndrome | 3 | 61 | 59 | 41.00 |
| Peptic Ulcer | 41 | 44 | 39 | 41.33 |
| Coronary Artery Disease | 56 | 8 | 62 | 42.00 |
| Race/Ethnicity: Unknown | 50 | 22 | 55 | 42.33 |
| Moderate to Severe Liver Disease | 27 | 53 | 48 | 42.67 |
| Tuberculosis | 20 | 60 | 50 | 43.33 |
| Malignant Cancer | 54 | 32 | 46 | 44.00 |
| Congestive Heart Failure | 46 | 36 | 52 | 44.67 |
| Mild Liver Disease | 43 | 47 | 45 | 45.00 |
| HIV | 30 | 51 | 58 | 46.33 |
| Obesity | 55 | 41 | 44 | 46.67 |
| Cerebrovascular Disease | 49 | 54 | 47 | 50.00 |
| Sickle Cell Disease | 45 | 59 | 51 | 51.67 |
| Thalassemia | 39 | 58 | 60 | 52.33 |
| AKI during COVID-associated Hospitalization | 57 | 50 | 54 | 53.67 |
| Race/Ethnicity: Other race NH | 59 | 55 | 53 | 55.67 |

**eTable 7. Comparison of Feature Importance Across Models for Unrestricted Sample including SDOH variables (PASC defined as U09.9 or Long-COVID Clinic Visit)**

| features | Logistic Regression | Random Forest | XGBoost | Mean Rank |
| --- | --- | --- | --- | --- |
| Hospitalization Extended Stay (31+ days) | 2 | 19 | 1 | 7.33 |
| MDs per 1000 residents: Low (<1.91%) | ref. | 12 | 6 | 9.00 |
| Age 50-59 | 5 | 14 | 12 | 10.33 |
| COVID-associated Hospitalization | 4 | 23 | 4 | 10.33 |
| Hospitalization Long Stay (8-30 days) | 8 | 27 | 2 | 12.33 |
| Race/Ethnicity: White NH | ref. | 7 | 18 | 12.50 |
| COVID Diagnosis during COVID-associated Hospitalization | 11 | 26 | 3 | 13.33 |
| Public health Insurance for ages 19-64: Low (<13%) | ref. | 13 | 14 | 13.50 |
| MDs per 1000 residents: High (>3.61%) | 30 | 8 | 8 | 15.33 |
| Age 18-29 | ref. | 21 | 10 | 15.50 |
| Chronic Lung Disease | 17 | 17 | 19 | 17.67 |
| COVID Treatment: Mechanical Ventilation | 23 | 28 | 7 | 19.33 |
| Depression | 19 | 31 | 11 | 20.33 |
| Female | 25 | 1 | 35 | 20.33 |
| MDs per 1000 residents: medium (1.91-3.61%) | 36 | 3 | 28 | 22.33 |
| College Degree low (<19%) | ref. | 10 | 39 | 24.50 |
| Substance Abuse | 15 | 40 | 21 | 25.33 |
| Male | ref. | 2 | 49 | 25.50 |
| Age 40-49 | 6 | 5 | 68 | 26.33 |
| Age 70-79 | 10 | 25 | 44 | 26.33 |
| COVID-associated ED Visit | 40 | 34 | 5 | 26.33 |
| Age 60-69 | 7 | 18 | 55 | 26.67 |
| Race/Ethnicity: Hispanic | 31 | 24 | 26 | 27.00 |
| Psychosis | 9 | 46 | 27 | 27.33 |
| Race/Ethnicity: Black NH | 24 | 22 | 38 | 28.00 |
| COVID Treatment: Remdesivir | 32 | 45 | 9 | 28.67 |
| Age 30-39 | 12 | 11 | 64 | 29.00 |
| Obesity | 41 | 38 | 13 | 30.67 |
| Age 80-89 | 21 | 44 | 29 | 31.33 |
| Systemic Corticosteroids | 29 | 50 | 17 | 32.00 |
| Households with Income below poverty: low (<11%) | ref. | 6 | 61 | 33.50 |
| College Degree high (>25%) | 57 | 15 | 32 | 34.67 |
| Tobacco Smoker | 27 | 37 | 40 | 34.67 |
| COVID Treatment: Vasopressors | 58 | 33 | 16 | 35.67 |
| Metastatic Solid Tumor Cancers | 18 | 60 | 30 | 36.00 |
| Public health Insurance for ages 19-64: Medium (13-18%) | 67 | 20 | 23 | 36.67 |
| College Degree medium (19-25%) | 51 | 4 | 57 | 37.33 |
| Peptic Ulcer | 38 | 53 | 22 | 37.67 |
| Cardiomyopathies | 33 | 48 | 34 | 38.33 |
| Households with Income below poverty: high (>15%) | 59 | 16 | 41 | 38.67 |
| Complicated Diabetes | 65 | 29 | 24 | 39.33 |
| AKI prior to COVID | 43 | 62 | 15 | 40.00 |
| Peripheral Vascular Disease | 44 | 51 | 25 | 40.00 |
| Uncomplicated Diabetes | 48 | 35 | 37 | 40.00 |
| Congestive Heart Failure | 46 | 55 | 20 | 40.33 |
| Race/Ethnicity: Unknown | 35 | 30 | 56 | 40.33 |
| Rheumatologic Disease | 34 | 57 | 31 | 40.67 |
| Dementia | 37 | 56 | 33 | 42.00 |
| COVID Treatment: ECMO | 28 | 66 | 36 | 43.33 |
| Hemiplegia or Paraplegia | 20 | 63 | 47 | 43.33 |
| Households with Income below poverty: medium (11-15%) | 50 | 9 | 72 | 43.67 |
| Public health Insurance for ages 19-64: High (>18%) | 53 | 32 | 48 | 44.33 |
| Down Syndrome | 3 | 72 | 62 | 45.67 |
| Heart Failure | 42 | 42 | 53 | 45.67 |
| Race/Ethnicity: Asian NH | 26 | 43 | 73 | 47.33 |
| Sepsis during COVID-associated Hospitalization | 47 | 52 | 45 | 48.00 |
| COVID Treatment: Corticosteroids | 63 | 41 | 42 | 48.67 |
| Hospitalization Medium Stay (3-7 days) | 54 | 39 | 54 | 49.00 |
| Moderate to Severe Liver Disease | 16 | 73 | 58 | 49.00 |
| Tuberculosis | 13 | 70 | 65 | 49.33 |
| Myocardial Infarction | 39 | 59 | 52 | 50.00 |
| Kidney Disease | 64 | 36 | 51 | 50.33 |
| Thalassemia | 14 | 64 | 74 | 50.67 |
| Age 90+ | 49 | 69 | 43 | 53.67 |
| Hospitalization Short Stay (0-2 days) | ref. | 49 | 59 | 54.00 |
| Sickle Cell Disease | 22 | 74 | 71 | 55.67 |
| Hypertension | 45 | 54 | 69 | 56.00 |
| Coronary Artery Disease | 55 | 68 | 46 | 56.33 |
| HIV | 56 | 67 | 50 | 57.67 |
| Malignant Cancer | 66 | 47 | 60 | 57.67 |
| Cerebrovascular Disease | 52 | 58 | 70 | 60.00 |
| Race/Ethnicity: Other race NH | 60 | 65 | 63 | 62.67 |
| AKI during COVID-associated Hospitalization | 62 | 61 | 67 | 63.33 |
| Mild Liver Disease | 61 | 71 | 66 | 66.00 |

**eTable 8. Cohort Characteristics for PASC cases defined by U09.9 or clinic visit for Hospitalized During COVID Index**

|  | PASC (N=3062) | Method 1  Unrestricted Controls  (N=15605) | Method 2  Restricted  controls  (N=15353) | Method 3  Most Restricted  controls  (N=12071) |
| --- | --- | --- | --- | --- |
| **Demographics** |  |  |  |  |
| Age |  |  |  |  |
| 18-29 | 132 (4.3%) | 1800 (11.5%) | 1931 (12.6%) | 2498 (20.7%) |
| 30-39 | 298 (9.7%) | 2140 (13.7%) | 2117 (13.8%) | 2040 (16.9%) |
| 40-49 | 535 (17.5%) | 2279 (14.6%) | 2050 (13.4%) | 1360 (11.3%) |
| 50-59 | 733 (23.9%) | 2780 (17.8%) | 2574 (16.8%) | 1326 (11.0%) |
| 60-69 | 735 (24.0%) | 2915 (18.7%) | 2808 (18.3%) | 1450 (12.0%) |
| 70-79 | 433 (14.1%) | 2146 (13.8%) | 2166 (14.1%) | 1481 (12.3%) |
| 80-89 | 171 (5.6%) | 1253 (8.0%) | 1346 (8.8%) | 1403 (11.6%) |
| 90+ | 24 (0.8%) | 218 (1.4%) | 264 (1.7%) | 390 (3.2%) |
| Sex |  |  |  |  |
| Female | 1627 (53.1%) | 8115 (52.0%) | 8260 (53.8%) | 6143 (50.9%) |
| Male | 1434 (46.8%) | 7488 (48.0%) | 7091 (46.2%) | 5927 (49.1%) |
| Race/Ethnicity |  |  |  |  |
| White non-Hispanic (NH) | 1878 (61.3%) | 8821 (56.5%) | 8982 (58.5%) | 6638 (55.0%) |
| Hispanic | 397 (13.0%) | 2410 (15.4%) | 2096 (13.7%) | 1875 (15.5%) |
| Black NH | 586 (19.1%) | 3161 (20.3%) | 3250 (21.2%) | 2666 (22.1%) |
| Asian NH | 57 (1.9%) | 368 (2.4%) | 357 (2.3%) | 286 (2.4%) |
| Other race NH | 22 (0.7%) | 134 (0.9%) | 101 (0.7%) | 87 (0.7%) |
| Unknown | 113 (3.7%) | 667 (4.3%) | 518 (3.4%) | 475 (3.9%) |
| **Comorbidities Prior to COVID Index Date** |  |  |  |  |
| AKI | 654 (21.4%) | 2987 (19.1%) | 3049 (19.9%) | 2119 (17.6%) |
| Cardiomyopathies | 147 (4.8%) | 679 (4.4%) | 780 (5.1%) | 457 (3.8%) |
| Cerebrovascular Disease | 226 (7.4%) | 1097 (7.0%) | 1304 (8.5%) | 956 (7.9%) |
| Chronic Lung Disease | 1113 (36.3%) | 3919 (25.1%) | 4008 (26.1%) | 2490 (20.6%) |
| Complicated Diabetes | 737 (24.1%) | 3164 (20.3%) | 3433 (22.4%) | 2139 (17.7%) |
| Congestive Heart Failure | 396 (12.9%) | 1738 (11.1%) | 1943 (12.7%) | 1293 (10.7%) |
| Coronary Artery Disease | 495 (16.2%) | 2153 (13.8%) | 2451 (16.0%) | 1637 (13.6%) |
| Dementia | 77 (2.5%) | 682 (4.4%) | 672 (4.4%) | 670 (5.6%) |
| Down Syndrome | <20 | <20 | <20 | <20 |
| Heart Failure | 490 (16.0%) | 2136 (13.7%) | 2320 (15.1%) | 1554 (12.9%) |
| Hemiplegia or Paraplegia | 36 (1.2%) | 254 (1.6%) | 319 (2.1%) | 220 (1.8%) |
| HIV | 21 (0.7%) | 103 (0.7%) | 156 (1.0%) | 104 (0.9%) |
| Hypertension | 1584 (51.7%) | 6684 (42.8%) | 7438 (48.4%) | 4941 (40.9%) |
| Kidney Disease | 836 (27.3%) | 3912 (25.1%) | 4135 (26.9%) | 2776 (23.0%) |
| Malignant Cancer | 415 (13.6%) | 1802 (11.5%) | 2172 (14.1%) | 1396 (11.6%) |
| Metastatic Solid Tumor Cancers | 55 (1.8%) | 339 (2.2%) | 432 (2.8%) | 239 (2.0%) |
| Mild Liver Disease | 97 (3.2%) | 531 (3.4%) | 626 (4.1%) | 388 (3.2%) |
| Moderate to Severe Liver Disease | 56 (1.8%) | 333 (2.1%) | 356 (2.3%) | 219 (1.8%) |
| Myocardial Infarction | 252 (8.2%) | 1348 (8.6%) | 1517 (9.9%) | 972 (8.1%) |
| Obesity | 2009 (65.6%) | 8284 (53.1%) | 8694 (56.6%) | 6206 (51.4%) |
| Peptic Ulcer | 140 (4.6%) | 503 (3.2%) | 581 (3.8%) | 379 (3.1%) |
| Peripheral Vascular Disease | 262 (8.6%) | 1018 (6.5%) | 1174 (7.6%) | 732 (6.1%) |
| Rheumatologic Disease | 155 (5.1%) | 474 (3.0%) | 528 (3.4%) | 239 (2.0%) |
| Sickle Cell Disease | <20 | 53 (0.3%) | 86 (0.6%) | 72 (0.6%) |
| Systemic Corticosteroids | 1879 (61.4%) | 7743 (49.6%) | 8090 (52.7%) | 5479 (45.4%) |
| Thalassemia | <20 | 39 (0.2%) | 61 (0.4%) | 57 (0.5%) |
| Tuberculosis | <20 | 46 (0.3%) | 67 (0.4%) | 36 (0.3%) |
| Uncomplicated Diabetes | 969 (31.6%) | 4228 (27.1%) | 4526 (29.5%) | 2986 (24.7%) |
| **Behavioral Health Indicators** |  |  |  |  |
| Depression | 742 (24.2%) | 2985 (19.1%) | 3462 (22.5%) | 2313 (19.2%) |
| Psychosis | 42 (1.4%) | 372 (2.4%) | 416 (2.7%) | 353 (2.9%) |
| Substance Abuse | 102 (3.3%) | 1003 (6.4%) | 1162 (7.6%) | 943 (7.8%) |
| Tobacco Smoker | 219 (7.2%) | 1628 (10.4%) | 1769 (11.5%) | 1253 (10.4%) |
| **Characteristics during Acute COVID Phase** |  |  |  |  |
| COVID Diagnosis during COVID-associated Hospitalization | 2889 (94.4%) | 13786 (88.3%) | 13264 (86.4%) | 10324 (85.5%) |
| COVID-associated Hospitalization | 3062 (100.0%) | 15605 (100.0%) | 15353 (100.0%) | 12071 (100.0%) |
| COVID-associated ED Visit | 608 (19.9%) | 2316 (14.8%) | 2586 (16.8%) | 1851 (15.3%) |
| Hospitalization Stay |  |  |  |  |
| Short Stay (0-2 days) | 603 (19.7%) | 5501 (35.3%) | 5447 (35.5%) | 4658 (38.6%) |
| Medium Stay (3-7 days) | 858 (28.0%) | 5430 (34.8%) | 5393 (35.1%) | 4131 (34.2%) |
| Long Stay (8-30 days) | 1017 (33.2%) | 3274 (21.0%) | 3076 (20.0%) | 2172 (18.0%) |
| Extended Stay (31+ days) | 443 (14.5%) | 572 (3.7%) | 461 (3.0%) | 299 (2.5%) |
| COVID Treatment |  |  |  |  |
| Corticosteroids | 2000 (65.3%) | 7734 (49.6%) | 7254 (47.2%) | 4947 (41.0%) |
| Remdesivir | 1390 (45.4%) | 4932 (31.6%) | 4417 (28.8%) | 2954 (24.5%) |
| Vasopressors | 588 (19.2%) | 1774 (11.4%) | 1856 (12.1%) | 1504 (12.5%) |
| ECMO | 67 (2.2%) | 52 (0.3%) | 22 (0.1%) | 21 (0.2%) |
| Mechanical Ventilation | 604 (19.7%) | 1130 (7.2%) | 858 (5.6%) | 551 (4.6%) |
| AKI during COVID-associated Hospitalization | 683 (22.3%) | 2497 (16.0%) | 2393 (15.6%) | 1692 (14.0%) |
| Sepsis during COVID-associated Hospitalization | 615 (20.1%) | 1974 (12.6%) | 1808 (11.8%) | 1248 (10.3%) |

**eTable 9. Cohort Characteristics for PASC cases defined by U09.9 or clinic visit for Not Hospitalized During COVID Index**

|  | PASC  (N=5232) | Method 1  Unrestricted Controls  (N=26245) | Method 2  Restricted  controls  (N=26160) | Method 3  Most Restricted  controls  (N=25616) |
| --- | --- | --- | --- | --- |
| **Demographics** |  |  |  |  |
| Age |  |  |  |  |
| 18-29 | 496 (9.5%) | 5697 (21.7%) | 5275 (20.2%) | 7149 (27.9%) |
| 30-39 | 930 (17.8%) | 5185 (19.8%) | 4832 (18.5%) | 4969 (19.4%) |
| 40-49 | 1207 (23.1%) | 4835 (18.4%) | 4542 (17.4%) | 4154 (16.2%) |
| 50-59 | 1192 (22.8%) | 4453 (17.0%) | 4637 (17.7%) | 3925 (15.3%) |
| 60-69 | 859 (16.4%) | 3389 (12.9%) | 3774 (14.4%) | 2552 (10.0%) |
| 70-79 | 406 (7.8%) | 1895 (7.2%) | 2172 (8.3%) | 1781 (7.0%) |
| 80-89 | 125 (2.4%) | 656 (2.5%) | 786 (3.0%) | 848 (3.3%) |
| 90+ | <20 | 112 (0.4%) | 122 (0.5%) | 195 (0.8%) |
| Sex |  |  |  |  |
| Female | 3581 (68.4%) | 14910 (56.8%) | 15670 (59.9%) | 14397 (56.2%) |
| Male | 1651 (31.6%) | 11294 (43.0%) | 10476 (40.0%) | 11206 (43.7%) |
| Race/Ethnicity |  |  |  |  |
| White non-Hispanic (NH) | 3815 (72.9%) | 17182 (65.5%) | 17877 (68.3%) | 17265 (67.4%) |
| Hispanic | 431 (8.2%) | 2769 (10.6%) | 2546 (9.7%) | 2721 (10.6%) |
| Black NH | 646 (12.3%) | 3651 (13.9%) | 3940 (15.1%) | 3674 (14.3%) |
| Asian NH | 77 (1.5%) | 534 (2.0%) | 582 (2.2%) | 625 (2.4%) |
| Other race NH | 32 (0.6%) | 190 (0.7%) | 181 (0.7%) | 186 (0.7%) |
| Unknown | 225 (4.3%) | 1884 (7.2%) | 996 (3.8%) | 1090 (4.3%) |
| **Comorbidities Prior to COVID Index Date** |  |  |  |  |
| AKI | 202 (3.9%) | 758 (2.9%) | 1066 (4.1%) | 776 (3.0%) |
| Cardiomyopathies | 79 (1.5%) | 333 (1.3%) | 470 (1.8%) | 318 (1.2%) |
| Cerebrovascular Disease | 164 (3.1%) | 615 (2.3%) | 860 (3.3%) | 663 (2.6%) |
| Chronic Lung Disease | 1289 (24.6%) | 3203 (12.2%) | 3925 (15.0%) | 3020 (11.8%) |
| Complicated Diabetes | 466 (8.9%) | 1657 (6.3%) | 2317 (8.9%) | 1650 (6.4%) |
| Congestive Heart Failure | 179 (3.4%) | 581 (2.2%) | 854 (3.3%) | 616 (2.4%) |
| Coronary Artery Disease | 338 (6.5%) | 1265 (4.8%) | 1761 (6.7%) | 1324 (5.2%) |
| Dementia | 74 (1.4%) | 243 (0.9%) | 366 (1.4%) | 320 (1.2%) |
| Down Syndrome | <20 | <20 | <20 | <20 |
| Heart Failure | 243 (4.6%) | 776 (3.0%) | 1095 (4.2%) | 798 (3.1%) |
| Hemiplegia or Paraplegia | 23 (0.4%) | 90 (0.3%) | 161 (0.6%) | 110 (0.4%) |
| HIV | 28 (0.5%) | 145 (0.6%) | 206 (0.8%) | 207 (0.8%) |
| Hypertension | 1765 (33.7%) | 6166 (23.5%) | 8258 (31.6%) | 6485 (25.3%) |
| Kidney Disease | 417 (8.0%) | 1478 (5.6%) | 2075 (7.9%) | 1537 (6.0%) |
| Malignant Cancer | 426 (8.1%) | 1635 (6.2%) | 2348 (9.0%) | 1760 (6.9%) |
| Metastatic Solid Tumor Cancers | 35 (0.7%) | 206 (0.8%) | 293 (1.1%) | 215 (0.8%) |
| Mild Liver Disease | 69 (1.3%) | 287 (1.1%) | 422 (1.6%) | 304 (1.2%) |
| Moderate to Severe Liver Disease | 24 (0.5%) | 114 (0.4%) | 179 (0.7%) | 115 (0.4%) |
| Myocardial Infarction | 138 (2.6%) | 552 (2.1%) | 804 (3.1%) | 616 (2.4%) |
| Obesity | 2657 (50.8%) | 9966 (38.0%) | 12238 (46.8%) | 10592 (41.3%) |
| Peptic Ulcer | 139 (2.7%) | 396 (1.5%) | 538 (2.1%) | 445 (1.7%) |
| Peripheral Vascular Disease | 142 (2.7%) | 458 (1.7%) | 683 (2.6%) | 471 (1.8%) |
| Rheumatologic Disease | 190 (3.6%) | 452 (1.7%) | 614 (2.3%) | 430 (1.7%) |
| Sickle Cell Disease | <20 | 20 (0.1%) | 46 (0.2%) | 41 (0.2%) |
| Systemic Corticosteroids | 2431 (46.5%) | 8019 (30.6%) | 10054 (38.4%) | 8365 (32.7%) |
| Thalassemia | <20 | 40 (0.2%) | 65 (0.2%) | 48 (0.2%) |
| Tuberculosis | <20 | 53 (0.2%) | 52 (0.2%) | 41 (0.2%) |
| Uncomplicated Diabetes | 729 (13.9%) | 2821 (10.7%) | 3810 (14.6%) | 2899 (11.3%) |
| **Behavioral Health Indicators** |  |  |  |  |
| Depression | 1314 (25.1%) | 3376 (12.9%) | 4622 (17.7%) | 3688 (14.4%) |
| Psychosis | 23 (0.4%) | 155 (0.6%) | 229 (0.9%) | 194 (0.8%) |
| Substance Abuse | 101 (1.9%) | 628 (2.4%) | 804 (3.1%) | 699 (2.7%) |
| Tobacco Smoker | 297 (5.7%) | 1452 (5.5%) | 1751 (6.7%) | 1577 (6.2%) |
| **Characteristics during Acute COVID Phase** |  |  |  |  |
| COVID-associated ED Visit | 945 (18.1%) | 4021 (15.3%) | 3559 (13.6%) | 3633 (14.2%) |

**eTable 10. PASC Risk Factors from Logistic Regression including SDoH (PASC defined as U09.9 or long-COVID visit)Not Hospitalized during COVID Index**

|  | Method 1  Unrestricted Controls  (N=28473) | Method 2  Restricted  controls  (N=28408) | Method 3  Most Restricted  controls  (N=27817) |
| --- | --- | --- | --- |
| **Demographics** |  |  |  |
| Age |  |  |  |
| 18-29 | REF | REF | REF |
| 30-39 | 1.91 (1.69-2.17) | 2.1 (1.85-2.38) | 2.73 (2.41-3.09) |
| 40-49 | 2.57 (2.28-2.9) | 2.98 (2.64-3.37) | 4.38 (3.87-4.94) |
| 50-59 | 2.65 (2.35-3.0) | 2.97 (2.63-3.37) | 4.61 (4.07-5.23) |
| 60-69 | 2.45 (2.14-2.8) | 2.67 (2.34-3.05) | 5.22 (4.55-5.99) |
| 70-79 | 1.88 (1.59-2.21) | 2.1 (1.78-2.47) | 3.14 (2.66-3.7) |
| 80-89 | 1.64 (1.29-2.09) | 1.83 (1.44-2.32) | 1.98 (1.55-2.53) |
| 90+ | 0.94 (0.5-1.75) | 1.14 (0.61-2.11) | 0.79 (0.43-1.46) |
| Sex |  |  |  |
| Male or Unknown Sex | REF | REF | REF |
| Female | 1.44 (1.34-1.55) | 1.34 (1.25-1.43) | 1.71 (1.59-1.84) |
| Race/Ethnicity |  |  |  |
| White NH | REF | REF | REF |
| Hispanic | 0.8 (0.71-0.9) | 0.85 (0.76-0.96) | 0.85 (0.75-0.96) |
| Black NH | 0.69 (0.62-0.77) | 0.69 (0.63-0.77) | 0.69 (0.62-0.77) |
| Asian NH | 0.75 (0.58-0.97) | 0.63 (0.49-0.81) | 0.61 (0.46-0.79) |
| Other race NH | 1.0 (0.64-1.56) | 1.07 (0.69-1.65) | 1.29 (0.82-2.04) |
| Unknown | 0.75 (0.64-0.88) | 1.19 (1.01-1.4) | 1.12 (0.94-1.33) |
| **Comorbidities Prior to COVID Index Date** |  |  |  |
| AKI | 0.83 (0.65-1.05) | 0.91 (0.72-1.14) | 0.78 (0.61-1.0) |
| Cardiomyopathies | 0.73 (0.58-0.97) | 0.68 (0.51-0.92) | 0.82 (0.59-1.12) |
| Cerebrovascular Disease | 0.91 (0.74-1.11) | 0.9 (0.74-1.1) | 0.91 (0.74-1.13) |
| Chronic Lung Disease | 1.77 (1.63-1.93) | 1.77 (1.63-1.92) | 2.03 (1.86-2.21) |
| Complicated Diabetes | 1.12 (0.94-1.34) | 1.14 (0.96-1.36) | 1.2 (0.99-1.44) |
| Congestive Heart Failure | 1.0 (0.69-1.45) | 0.93 (0.65-1.32) | 1.06 (0.71-1.57) |
| Coronary Artery Disease | 1.03 (0.88-1.21) | 0.97 (0.83-1.13) | 1.05 (0.89-1.24) |
| Dementia | 1.19 (0.87-1.61) | 0.98 (0.74-1.31) | 0.96 (0.72-1.3) |
| Down Syndrome | 2.25 (0.19-26.79) | 4.4 (0.27-72.5) | 1.22 (0.13-11.24) |
| Heart Failure | 1.16 (0.84-1.6) | 1.21 (0.89-1.65) | 1.15 (0.82-1.63) |
| Hemiplegia or Paraplegia | 0.84 (0.5-1.41) | 0.64 (0.39-1.05) | 0.64 (0.38-1.06) |
| HIV | 0.83 (0.53-1.32) | 0.64 (0.41-1.01) | 0.6 (0.38-0.95) |
| Hypertension | 1.14 (1.05-1.24) | 0.95 (0.88-1.04) | 1.0 (0.91-1.09) |
| Kidney Disease | 1.14 (0.95-1.36) | 1.06 (0.89-1.25) | 1.18 (0.98-1.41) |
| Malignant Cancer | 0.96 (0.84-1.1) | 0.82 (0.72-0.93) | 0.94 (0.82-1.07) |
| Metastatic Solid Tumor Cancers | 0.57 (0.38-0.85) | 0.6 (0.41-0.89) | 0.6 (0.4-0.89) |
| Mild Liver Disease | 1.0 (0.72-1.39) | 0.86 (0.63-1.18) | 0.96 (0.69-1.34) |
| Moderate to Severe Liver Disease | 0.64 (0.36-1.11) | 0.63 (0.37-1.07) | 0.59 (0.33-1.06) |
| Myocardial Infarction | 0.77 (0.6-0.97) | 0.78 (0.62-0.98) | 0.66 (0.52-0.85) |
| Obesity | 1.18 (1.1-1.26) | 0.97 (0.91-1.04) | 1.06 (0.99-1.14) |
| Peptic Ulcer | 1.22 (0.98-1.52) | 1.15 (0.94-1.42) | 1.11 (0.89-1.39) |
| Peripheral Vascular Disease | 1.16 (0.93-1.45) | 1.06 (0.86-1.31) | 1.27 (1.02-1.6) |
| Rheumatologic Disease | 1.25 (1.03-1.5) | 1.21 (1.01-1.44) | 1.4 (1.15-1.7) |
| Sickle Cell Disease | 0.7 (0.19-2.52) | 0.41 (0.12-1.36) | 0.48 (0.14-1.67) |
| Systemic Corticosteroids | 1.35 (1.25-1.44) | 1.14 (1.07-1.23) | 1.3 (1.21-1.4) |
| Thalassemia | 1.95 (1.03-3.72) | 1.48 (0.81-2.68) | 1.72 (0.91-3.22) |
| Tuberculosis | 1.02 (0.53-1.95) | 1.28 (0.67-2.47) | 1.84 (0.91-3.71) |
| Uncomplicated Diabetes | 0.8 (0.69-0.92) | 0.77 (0.67-0.89) | 0.8 (0.69-0.93) |
| **Behavioral Health** |  |  |  |
| Depression | 1.66 (1.52-1.8) | 1.41 (1.3-1.53) | 1.63 (1.5-1.78) |
| Psychosis | 0.55 (0.33-0.91) | 0.52 (0.32-0.86) | 0.47 (0.28-0.78) |
| Substance Abuse | 0.61 (0.47-0.78) | 0.61 (0.48-0.77) | 0.64 (0.5-0.83) |
| Tobacco Smoker | 0.77 (0.67-0.89) | 0.75 (0.65-0.86) | 0.72 (0.63-0.84) |
| **Characteristics of Index COVID "Acute Phase"** |  |  |  |
| COVID-associated ED Visit | 1.2 (1.1-1.31) | 1.46 (1.34-1.6) | 1.43 (1.3-1.56) |
| **Social Determinants of Health** |  |  |  |
| Households with Income below poverty: medium (11-15%) | 0.86 (0.79-0.94) | 0.77 (0.71-0.84) | 0.76 (0.7-0.83) |
| Households with Income below poverty: high (>15%) | 0.92 (0.84-1.0) | 0.94 (0.86-1.03) | 0.9 (0.82-0.99) |
| College Degree medium (19-25%) | 0.97 (0.87-1.09) | 0.98 (0.88-1.1) | 0.92 (0.82-1.04) |
| College Degree high (>25%) | 1.0 (0.87-1.15) | 0.97 (0.85-1.11) | 0.93 (0.81-1.08) |
| Public health Insurance for ages 19-64: Medium (13-18%) | 1.04 (0.96-1.12) | 1.05 (0.98-1.14) | 1.06 (0.98-1.15) |
| Public health Insurance for ages 19-64: High (>18%) | 0.94 (0.86-1.03) | 0.9 (0.83-0.99) | 0.9 (0.82-0.99) |
| MDs per 1000 residents: medium (1.91-3.61%) | 1.22 (1.09-1.37) | 1.15 (1.02-1.29) | 1.22 (1.08-1.37) |
| MDs per 1000 residents: High (>3.61%) | 1.18 (1.05-1.34) | 1.04 (0.92-1.17) | 1.17 (1.03-1.33) |

**eTable 11. PASC Risk Factors from Logistic Regression including SDoH (PASC defined as U09.9 or long-COVID visit) Hospitalized during COVID Index**

|  | Method 1  Unrestricted Controls  (N=16394) | Method 2  Restricted  controls  (N=16110) | Method 3  Most Restricted  controls  (N=12395) |
| --- | --- | --- | --- |
| **Demographics** |  |  |  |
| Age |  |  |  |
| 18-29 | REF | REF | REF |
| 30-39 | 1.73 (1.37-2.18) | 1.91 (1.51-2.42) | 2.77 (2.1-3.64) |
| 40-49 | 2.45 (1.96-3.05) | 3.07 (2.46-3.85) | 6.83 (5.29-8.81) |
| 50-59 | 2.57 (2.07-3.19) | 3.19 (2.56-3.97) | 8.79 (6.77-11.41) |
| 60-69 | 2.23 (1.79-2.78) | 2.77 (2.22-3.47) | 7.02 (5.37-9.17) |
| 70-79 | 1.75 (1.39-2.22) | 2.22 (1.75-2.82) | 3.86 (2.89-5.14) |
| 80-89 | 1.24 (0.93-1.64) | 1.38 (1.04-1.85) | 1.53 (1.09-2.14) |
| 90+ | 1.07 (0.64-1.8) | 0.98 (0.58-1.67) | 0.79 (0.44-1.42) |
| Sex |  |  |  |
| Male or Unknown Sex | REF | REF | REF |
| Female | 1.07 (0.98-1.18) | 1.06 (0.96-1.16) | 1.56 (1.38-1.76) |
| Race/Ethnicity |  |  |  |
| White NH | REF | REF | REF |
| Hispanic | 0.74 (0.64-0.85) | 0.89 (0.77-1.02) | 0.8 (0.68-0.95) |
| Black NH | 0.9 (0.79-1.02) | 0.9 (0.79-1.03) | 0.81 (0.71-0.93) |
| Asian NH | 0.78 (0.57-1.06) | 0.77 (0.56-1.06) | 0.68 (0.46-0.99) |
| Other race NH | 0.91 (0.53-1.56) | 1.3 (0.75-2.27) | 0.73 (0.34-1.58) |
| Unknown | 0.84 (0.65-1.08) | 1.05 (0.81-1.37) | 0.9 (0.66-1.23) |
| **Comorbidities Prior to COVID Index Date** |  |  |  |
| AKI | 0.89 (0.72-1.1) | 0.94 (0.75-1.16) | 0.84 (0.64-1.1) |
| Cardiomyopathies | 0.94 (0.75-1.19) | 0.93 (0.74-1.17) | 1.2 (0.9-1.58) |
| Cerebrovascular Disease | 1.07 (0.89-1.29) | 0.9 (0.75-1.08) | 0.81 (0.65-1.02) |
| Chronic Lung Disease | 1.21 (1.1-1.35) | 1.29 (1.16-1.43) | 1.57 (1.38-1.79) |
| Complicated Diabetes | 0.86 (0.72-1.03) | 0.88 (0.73-1.06) | 0.91 (0.72-1.15) |
| Congestive Heart Failure | 1.02 (0.77-1.36) | 0.95 (0.71-1.27) | 1.04 (0.72-1.5) |
| Coronary Artery Disease | 1.17 (1.01-1.36) | 1.17 (1.01-1.36) | 1.28 (1.07-1.54) |
| Dementia | 0.61 (0.46-0.81) | 0.68 (0.51-0.9) | 0.58 (0.42-0.81) |
| Down Syndrome | 2.61 (0.54-12.6) | 0.47 (0.08-2.69) | 0.28 (0.02-4.28) |
| Heart Failure | 0.99 (0.76-1.28) | 1.06 (0.81-1.38) | 0.94 (0.68-1.3) |
| Hemiplegia or Paraplegia | 0.48 (0.31-0.74) | 0.38 (0.24-0.59) | 0.28 (0.16-0.49) |
| HIV | 1.04 (0.61-1.77) | 0.7 (0.4-1.2) | 0.78 (0.41-1.49) |
| Hypertension | 1.19 (1.07-1.32) | 0.98 (0.88-1.09) | 1.1 (0.97-1.26) |
| Kidney Disease | 0.87 (0.72-1.04) | 0.84 (0.7-1.01) | 0.98 (0.78-1.22) |
| Malignant Cancer | 1.12 (0.96-1.29) | 1.02 (0.88-1.18) | 1.17 (0.98-1.41) |
| Metastatic Solid Tumor Cancers | 0.72 (0.51-1.02) | 0.67 (0.47-0.94) | 0.69 (0.45-1.04) |
| Mild Liver Disease | 0.99 (0.75-1.3) | 0.89 (0.67-1.17) | 0.94 (0.67-1.32) |
| Moderate to Severe Liver Disease | 0.65 (0.45-0.93) | 0.68 (0.47-0.99) | 0.63 (0.4-1.0) |
| Myocardial Infarction | 0.79 (0.66-0.95) | 0.73 (0.61-0.88) | 0.63 (0.48-0.81) |
| Obesity | 1.33 (1.21-1.47) | 1.22 (1.1-1.35) | 1.28 (1.12-1.46) |
| Peptic Ulcer | 1.26 (1.01-1.58) | 1.26 (1.01-1.58) | 1.11 (0.83-1.49) |
| Peripheral Vascular Disease | 1.17 (0.98-1.4) | 1.18 (0.98-1.41) | 1.37 (1.07-1.75) |
| Rheumatologic Disease | 1.44 (1.16-1.79) | 1.34 (1.09-1.64) | 1.83 (1.38-2.43) |
| Sickle Cell Disease | 2.57 (1.29-5.14) | 1.74 (0.89-3.38) | 1.86 (0.86-4.01) |
| Systemic Corticosteroids | 1.22 (1.1-1.35) | 1.17 (1.06-1.29) | 1.23 (1.09-1.39) |
| Thalassemia | 0.39 (0.13-1.21) | 0.5 (0.17-1.49) | 0.43 (0.12-1.53) |
| Tuberculosis | 1.74 (0.86-3.52) | 1.21 (0.62-2.37) | 1.71 (0.75-3.86) |
| Uncomplicated Diabetes | 1.01 (0.86-1.18) | 0.97 (0.82-1.14) | 1.0 (0.82-1.23) |
| **Behavioral Health** |  |  |  |
| Depression | 1.25 (1.12-1.4) | 1.22 (1.09-1.36) | 1.38 (1.2-1.58) |
| Psychosis | 0.56 (0.38-0.83) | 0.53 (0.36-0.79) | 0.43 (0.27-0.69) |
| Substance Abuse | 0.62 (0.48-0.79) | 0.53 (0.41-0.67) | 0.4 (0.3-0.55) |
| Tobacco Smoker | 0.67 (0.57-0.8) | 0.69 (0.58-0.82) | 0.62 (0.49-0.77) |
| **Characteristics of Index COVID "Acute Phase"** |  |  |  |
| COVID Diagnosis during COVID-associated Hospitalization | 1.81 (1.48-2.21) | 2.07 (1.68-2.54) | 1.94 (1.53-2.46) |
| COVID-associated Hospitalization | 0.19 (nan-nan) | 0.18 (nan-nan) | 0.12 (nan-nan) |
| COVID-associated ED Visit | 1.77 (1.58-1.99) | 1.52 (1.36-1.71) | 1.69 (1.46-1.95) |
| Hospitalization Stay |  |  |  |
| Not Hospitalized | REF | REF | REF |
| Hospitalization Short Stay (0-2 days) | 0.58 (0.45-0.75) | 0.58 (0.45-0.76) | 0.51 (0.38-0.69) |
| Hospitalization Medium Stay (3-7 days) | 0.69 (0.53-0.9) | 0.65 (0.5-0.86) | 0.56 (0.43-0.76) |
| Hospitalization Long Stay (8-30 days) | 1.33 (1.02-1.74) | 1.29 (0.98-1.7) | 1.26 (0.94-1.7) |
| Hospitalization Extended Stay (31+ days) | 2.65 (1.93-3.63) | 2.81 (2.03-3.9) | 3.03 (2.13-4.33) |
| COVID Treatment |  |  |  |
| Corticosteroids | 1.08 (0.97-1.22) | 1.17 (1.05-1.31) | 1.21 (1.05-1.4) |
| Remdesivir | 1.13 (1.02-1.26) | 1.25 (1.12-1.39) | 1.38 (1.2-1.58) |
| ECMO | 2.28 (1.47-3.53) | 4.01 (2.29-7.01) | 2.54 (1.3-4.95) |
| Mechanical Ventilation | 1.43 (1.21-1.69) | 1.77 (1.49-2.11) | 1.69 (1.35-2.11) |
| Vasopressors | 0.94 (0.81-1.09) | 0.8 (0.69-0.93) | 0.76 (0.63-0.92) |
| AKI during COVID-associated Hospitalization | 1.07 (0.91-1.26) | 1.1 (0.93-1.29) | 1.06 (0.86-1.3) |
| Sepsis during COVID-associated Hospitalization | 0.94 (0.82-1.07) | 0.95 (0.83-1.09) | 0.93 (0.79-1.1) |
| **Social Determinants of Health** |  |  |  |
| Households with Income below poverty: medium (11-15%) | 1.04 (0.92-1.18) | 0.99 (0.88-1.12) | 1.0 (0.89-1.14) |
| Households with Income below poverty: high (>15%) | 1.0 (0.88-1.13) | 1.06 (0.93-1.21) | 1.07 (0.97-1.17) |
| College Degree medium (19-25%) | 0.86 (0.74-0.99) | 0.91 (0.8-1.04) | 0.88 (nan-nan) |
| College Degree high (>25%) | 0.84 (0.7-1.0) | 0.83 (0.7-0.97) | 0.77 (nan-nan) |
| Public health Insurance for ages 19-64: Medium (13-18%) | 0.9 (0.81-0.99) | 0.89 (0.8-0.98) | 0.84 (0.74-0.95) |
| Public health Insurance for ages 19-64: High (>18%) | 0.87 (0.78-0.98) | 0.84 (nan-nan) | 0.8 (0.69-0.93) |
| MDs per 1000 residents: medium (1.91-3.61%) | 1.27 (1.09-1.47) | 1.16 (1.01-1.35) | 1.33 (1.17-1.51) |
| MDs per 1000 residents: High (>3.61%) | 1.54 (1.33-1.79) | 1.36 (1.17-1.58) | 1.54 (1.38-1.71) |

**eTable 12. Comparison of Feature Importance Across Models for Hospitalized during Index COVID for Unrestricted Sample including SDOH variables (PASC defined as U09.9 or Long-COVID Clinic Visit)**

| features | Logistic Regression | Random Forest | XGBoost | Mean Rank |
| --- | --- | --- | --- | --- |
| Hospitalization Extended Stay (31+ days) | 2 | 1 | 1 | 1.33 |
| College Degree low (<19%) | ref. | 3 | 10 | 6.50 |
| COVID-associated ED Visit | 14 | 6 | 2 | 7.33 |
| Male | ref. | 5 | 13 | 9.00 |
| Age 50-59 | 4 | 23 | 7 | 11.33 |
| Hospitalization Short Stay (0-2 days) | ref. | 24 | 3 | 13.50 |
| MDs per 1000 residents: High (>3.61%) | 20 | 13 | 8 | 13.67 |
| COVID Treatment: ECMO | 8 | 25 | 9 | 14.00 |
| Age 40-49 | 7 | 32 | 5 | 14.67 |
| COVID Diagnosis during COVID-associated Hospitalization | 17 | 28 | 17 | 20.67 |
| MDs per 1000 residents: medium (1.91-3.61%) | 29 | 12 | 23 | 21.33 |
| Substance Abuse | 19 | 35 | 12 | 22.00 |
| Age 60-69 | 9 | 7 | 51 | 22.33 |
| College Degree medium (19-25%) | 39 | 2 | 27 | 22.67 |
| Race/Ethnicity: White NH | ref. | 8 | 39 | 23.50 |
| Obesity | 27 | 29 | 15 | 23.67 |
| Chronic Lung Disease | 35 | 19 | 19 | 24.33 |
| COVID Treatment: Mechanical Ventilation | 24 | 47 | 4 | 25.00 |
| Hospitalization Long Stay (8-30 days) | 10 | 11 | 55 | 25.33 |
| Public health Insurance for ages 19-64: Low (<13%) | ref. | 31 | 20 | 25.50 |
| Tobacco Smoker | 22 | 41 | 14 | 25.67 |
| Hospitalization Medium Stay (3-7 days) | 47 | 26 | 6 | 26.33 |
| Female | 52 | 4 | 28 | 28.00 |
| MDs per 1000 residents: Low (<1.91%) | ref. | 9 | 47 | 28.00 |
| Age 30-39 | 15 | 22 | 48 | 28.33 |
| Age 70-79 | 13 | 39 | 34 | 28.67 |
| Race/Ethnicity: Hispanic | 26 | 15 | 45 | 28.67 |
| Depression | 32 | 30 | 25 | 29.00 |
| Kidney Disease | 43 | 38 | 11 | 30.67 |
| Households with Income below poverty: low (<11%) | ref. | 18 | 46 | 32.00 |
| COVID Treatment: Remdesivir | 45 | 34 | 18 | 32.33 |
| Race/Ethnicity: Black NH | 49 | 14 | 41 | 34.67 |
| Age 18-29 | ref. | 40 | 32 | 36.00 |
| Households with Income below poverty: medium (11-15%) | 61 | 10 | 37 | 36.00 |
| COVID Treatment: Vasopressors | 59 | 36 | 16 | 37.00 |
| Hypertension | 38 | 44 | 29 | 37.00 |
| College Degree high (>25%) | 36 | 20 | 56 | 37.33 |
| COVID Treatment: Corticosteroids | 53 | 16 | 43 | 37.33 |
| Peptic Ulcer | 31 | 62 | 21 | 38.00 |
| Households with Income below poverty: high (>15%) | 62 | 17 | 38 | 39.00 |
| Moderate to Severe Liver Disease | 21 | 56 | 40 | 39.00 |
| Age 80-89 | 33 | 43 | 42 | 39.33 |
| Peripheral Vascular Disease | 41 | 46 | 33 | 40.00 |
| Public health Insurance for ages 19-64: Medium (13-18%) | 50 | 21 | 49 | 40.00 |
| Race/Ethnicity: Unknown | 37 | 55 | 31 | 41.00 |
| Public health Insurance for ages 19-64: High (>18%) | 44 | 27 | 58 | 43.00 |
| Sickle Cell Disease | 6 | 70 | 54 | 43.33 |
| Systemic Corticosteroids | 34 | 33 | 63 | 43.33 |
| Uncomplicated Diabetes | 66 | 42 | 22 | 43.33 |
| AKI prior to COVID | 48 | 53 | 30 | 43.67 |
| Hemiplegia or Paraplegia | 11 | 61 | 61 | 44.33 |
| Thalassemia | 5 | 66 | 62 | 44.33 |
| AKI during COVID-associated Hospitalization | 54 | 54 | 26 | 44.67 |
| Congestive Heart Failure | 63 | 37 | 36 | 45.33 |
| Dementia | 18 | 57 | 64 | 46.33 |
| Down Syndrome | 3 | 71 | 65 | 46.33 |
| Psychosis | 12 | 59 | 68 | 46.33 |
| Rheumatologic Disease | 23 | 60 | 57 | 46.67 |
| Coronary Artery Disease | 40 | 49 | 52 | 47.00 |
| Sepsis during COVID-associated Hospitalization | 58 | 65 | 24 | 49.00 |
| Metastatic Solid Tumor Cancers | 25 | 58 | 66 | 49.67 |
| Myocardial Infarction | 30 | 50 | 70 | 50.00 |
| Race/Ethnicity: Asian NH | 28 | 51 | 73 | 50.67 |
| Cerebrovascular Disease | 55 | 48 | 50 | 51.00 |
| Complicated Diabetes | 42 | 52 | 60 | 51.33 |
| Heart Failure | 65 | 45 | 44 | 51.33 |
| Tuberculosis | 16 | 69 | 71 | 52.00 |
| Mild Liver Disease | 64 | 73 | 35 | 57.33 |
| Age 90+ | 56 | 68 | 53 | 59.00 |
| Malignant Cancer | 46 | 72 | 59 | 59.00 |
| Race/Ethnicity: Other race NH | 51 | 64 | 67 | 60.67 |
| Cardiomyopathies | 57 | 67 | 69 | 64.33 |
| HIV | 60 | 63 | 72 | 65.00 |
| COVID-associated Hospitalization | ref. | 74 | 74 | 74.00 |

**eTable 13. Comparison of Feature Importance Across Models for Not-Hospitalized during Index COVID for Unrestricted Sample including SDOH variables**

| features | Logistic Regression | Random Forest | XGBoost | Mean Rank |
| --- | --- | --- | --- | --- |
| Male | ref. | 1 | 10 | 5.50 |
| Age 40-49 | 3 | 12 | 2 | 5.67 |
| Female | 17 | 2 | 4 | 7.67 |
| Race/Ethnicity: White NH | ref. | 8 | 8 | 8.00 |
| Chronic Lung Disease | 10 | 22 | 3 | 11.67 |
| Depression | 12 | 32 | 1 | 15.00 |
| MDs per 1000 residents: Low (<1.91%) | ref. | 10 | 22 | 16.00 |
| Households with Income below poverty: medium (11-15%) | 39 | 5 | 9 | 17.67 |
| Households with Income below poverty: low (<11%) | ref. | 7 | 29 | 18.00 |
| Age 30-39 | 7 | 19 | 31 | 19.00 |
| Uncomplicated Diabetes | 25 | 29 | 5 | 19.67 |
| COVID-associated ED Visit | 32 | 21 | 7 | 20.00 |
| Race/Ethnicity: Unknown | 22 | 24 | 15 | 20.33 |
| Age 60-69 | 4 | 17 | 41 | 20.67 |
| Systemic Corticosteroids | 20 | 27 | 17 | 21.33 |
| Age 50-59 | 2 | 15 | 48 | 21.67 |
| College Degree low (<19%) | ref. | 11 | 37 | 24.00 |
| College Degree medium (19-25%) | 50 | 4 | 18 | 24.00 |
| Psychosis | 9 | 40 | 24 | 24.33 |
| Hypertension | 40 | 25 | 12 | 25.67 |
| MDs per 1000 residents: High (>3.61%) | 35 | 3 | 40 | 26.00 |
| Public health Insurance for ages 19-64: Medium (13-18%) | 48 | 18 | 13 | 26.33 |
| Substance Abuse | 13 | 46 | 20 | 26.33 |
| Rheumatologic Disease | 27 | 42 | 11 | 26.67 |
| Age 70-79 | 8 | 26 | 47 | 27.00 |
| Age 80-89 | 14 | 38 | 32 | 28.00 |
| Coronary Artery Disease | 49 | 31 | 6 | 28.67 |
| Peptic Ulcer | 29 | 36 | 21 | 28.67 |
| Metastatic Solid Tumor Cancers | 11 | 37 | 39 | 29.00 |
| Race/Ethnicity: Black NH | 16 | 20 | 51 | 29.00 |
| MDs per 1000 residents: medium (1.91-3.61%) | 28 | 9 | 54 | 30.33 |
| Tobacco Smoker | 24 | 45 | 23 | 30.67 |
| Complicated Diabetes | 42 | 28 | 25 | 31.67 |
| Obesity | 36 | 34 | 26 | 32.00 |
| Public health Insurance for ages 19-64: Low (<13%) | ref. | 14 | 50 | 32.00 |
| Heart Failure | 37 | 41 | 19 | 32.33 |
| Race/Ethnicity: Hispanic | 26 | 23 | 49 | 32.67 |
| Dementia | 34 | 50 | 16 | 33.33 |
| Moderate to Severe Liver Disease | 15 | 51 | 35 | 33.67 |
| College Degree high (>25%) | 55 | 13 | 36 | 34.67 |
| Kidney Disease | 41 | 30 | 34 | 35.00 |
| Peripheral Vascular Disease | 38 | 35 | 33 | 35.33 |
| Households with Income below poverty: high (>15%) | 44 | 6 | 59 | 36.33 |
| Age 18-29 | ref. | 16 | 57 | 36.50 |
| Race/Ethnicity: Asian NH | 21 | 33 | 56 | 36.67 |
| Hemiplegia or Paraplegia | 33 | 52 | 27 | 37.33 |
| AKI prior to COVID | 30 | 39 | 46 | 38.33 |
| Cerebrovascular Disease | 43 | 61 | 14 | 39.33 |
| Thalassemia | 6 | 58 | 55 | 39.67 |
| Cardiomyopathies | 19 | 59 | 42 | 40.00 |
| Public health Insurance for ages 19-64: High (>18%) | 46 | 48 | 28 | 40.67 |
| Myocardial Infarction | 23 | 47 | 53 | 41.00 |
| Down Syndrome | 5 | 60 | 60 | 41.67 |
| Sickle Cell Disease | 18 | 57 | 58 | 44.33 |
| HIV | 31 | 53 | 52 | 45.33 |
| Mild Liver Disease | 53 | 54 | 30 | 45.67 |
| Congestive Heart Failure | 52 | 44 | 44 | 46.67 |
| Race/Ethnicity: Other race NH | 54 | 49 | 38 | 47.00 |
| Age 90+ | 45 | 55 | 45 | 48.33 |
| Tuberculosis | 51 | 56 | 43 | 50.00 |
| Malignant Cancer | 47 | 43 | 61 | 50.33 |

**Other Definitions of Long-COVID**

**eTable 14. Characteristics of Cohorts for U09.9 only**

|  | U09.9 Only  (N=7512) | Method 1  Unrestricted controls (N=37575) | Method 2  Restricted controls (N=37560) | Method 3  Most restricted  controls (N=37560) |
| --- | --- | --- | --- | --- |
| **Demographics** |  |  |  |  |
| Age |  |  |  |  |
| Age 18-29 | 577 (7.7%) | 7633 (20.3%) | 7488 (19.9%) | 6890 (18.3%) |
| Age 30-39 | 1090 (14.5%) | 7206 (19.2%) | 7030 (18.7%) | 6681 (17.8%) |
| Age 40-49 | 1580 (21.0%) | 6698 (17.8%) | 6246 (16.6%) | 6482 (17.3%) |
| Age 50-59 | 1738 (23.1%) | 6235 (16.6%) | 6346 (16.9%) | 6534 (17.4%) |
| Age 60-69 | 1450 (19.3%) | 5235 (13.9%) | 5269 (14.0%) | 5838 (15.5%) |
| Age 70-79 | 766 (10.2%) | 3094 (8.2%) | 3450 (9.2%) | 3457 (9.2%) |
| Age 80-89 | 268 (3.6%) | 1220 (3.2%) | 1425 (3.8%) | 1393 (3.7%) |
| Age 90+ | 43 (0.6%) | 254 (0.7%) | 306 (0.8%) | 285 (0.8%) |
| Sex |  |  |  |  |
| Female | 4752 (63.3%) | 20826 (55.4%) | 21974 (58.5%) | 22483 (59.9%) |
| Male | 2759 (36.7%) | 16694 (44.4%) | 15567 (41.4%) | 15052 (40.1%) |
| Race |  |  |  |  |
| White non-Hispanic (NH) | 5301 (70.6%) | 24371 (64.9%) | 25307 (67.4%) | 25573 (68.1%) |
| Hispanic NH | 711 (9.5%) | 4217 (11.2%) | 3936 (10.5%) | 3783 (10.1%) |
| Black NH | 1035 (13.8%) | 5330 (14.2%) | 5771 (15.4%) | 5599 (14.9%) |
| Asian NH | 116 (1.5%) | 770 (2.0%) | 779 (2.1%) | 749 (2.0%) |
| Other race NH | 52 (0.7%) | 299 (0.8%) | 261 (0.7%) | 263 (0.7%) |
| Unknown | 282 (3.8%) | 2515 (6.7%) | 1435 (3.8%) | 1508 (4.0%) |
| **Comorbidities Prior to COVID Index Date** |  |  |  |  |
| AKI | 814 (10.8%) | 2056 (5.5%) | 2638 (7.0%) | 2626 (7.0%) |
| Cardiomyopathies | 208 (2.8%) | 648 (1.7%) | 933 (2.5%) | 913 (2.4%) |
| Cerebrovascular Disease | 364 (4.8%) | 1226 (3.3%) | 1718 (4.6%) | 1682 (4.5%) |
| Chronic Lung Disease | 2245 (29.9%) | 5248 (14.0%) | 6586 (17.5%) | 6642 (17.7%) |
| Complicated Diabetes | 1136 (15.1%) | 3276 (8.7%) | 4210 (11.2%) | 4285 (11.4%) |
| Congestive Heart Failure | 522 (6.9%) | 1424 (3.8%) | 1875 (5.0%) | 1886 (5.0%) |
| Coronary Artery Disease | 778 (10.4%) | 2459 (6.5%) | 3198 (8.5%) | 3246 (8.6%) |
| Dementia | 145 (1.9%) | 604 (1.6%) | 772 (2.1%) | 689 (1.8%) |
| Down Syndrome | <20 | <20 | <20 | <20 |
| Heart Failure | 669 (8.9%) | 1757 (4.7%) | 2310 (6.2%) | 2334 (6.2%) |
| Hemiplegia or Paraplegia | 58 (0.8%) | 235 (0.6%) | 379 (1.0%) | 309 (0.8%) |
| HIV | 41 (0.5%) | 203 (0.5%) | 262 (0.7%) | 289 (0.8%) |
| Hypertension | 3145 (41.9%) | 10101 (26.9%) | 12844 (34.2%) | 13026 (34.7%) |
| Kidney Disease | 1183 (15.7%) | 3313 (8.8%) | 4240 (11.3%) | 4250 (11.3%) |
| Malignant Cancer | 783 (10.4%) | 2588 (6.9%) | 3642 (9.7%) | 3717 (9.9%) |
| Metastatic Solid Tumor Cancers | 87 (1.2%) | 363 (1.0%) | 515 (1.4%) | 537 (1.4%) |
| Mild Liver Disease | 159 (2.1%) | 564 (1.5%) | 790 (2.1%) | 775 (2.1%) |
| Moderate to Severe Liver Disease | 77 (1.0%) | 259 (0.7%) | 380 (1.0%) | 371 (1.0%) |
| Myocardial Infarction | 371 (4.9%) | 1272 (3.4%) | 1630 (4.3%) | 1542 (4.1%) |
| Obesity | 4279 (57.0%) | 14822 (39.4%) | 17954 (47.8%) | 18008 (47.9%) |
| Peptic Ulcer | 270 (3.6%) | 712 (1.9%) | 958 (2.6%) | 934 (2.5%) |
| Perpheral Vascular Disease | 388 (5.2%) | 960 (2.6%) | 1395 (3.7%) | 1375 (3.7%) |
| Rheumatologic Disease | 334 (4.4%) | 749 (2.0%) | 945 (2.5%) | 1027 (2.7%) |
| Sickle Cell Disease | <20 | 42 (0.1%) | 99 (0.3%) | 85 (0.2%) |
| Systemic Corticosteroids | 4015 (53.4%) | 12627 (33.6%) | 15483 (41.2%) | 15526 (41.3%) |
| Thalassemia | <20 | 48 (0.1%) | 102 (0.3%) | 89 (0.2%) |
| Tuberculosis | 24 (0.3%) | 72 (0.2%) | 105 (0.3%) | 87 (0.2%) |
| Uncomplicated Diabetes | 1589 (21.2%) | 5088 (13.5%) | 6359 (16.9%) | 6452 (17.2%) |
| **Behavioral Health** |  |  |  |  |
| Depression | 1946 (25.9%) | 5395 (14.4%) | 7257 (19.3%) | 7430 (19.8%) |
| Psychosis | 61 (0.8%) | 350 (0.9%) | 460 (1.2%) | 435 (1.2%) |
| Substance Abuse | 196 (2.6%) | 1165 (3.1%) | 1592 (4.2%) | 1424 (3.8%) |
| Tobacco Smoker | 504 (6.7%) | 2515 (6.7%) | 3143 (8.4%) | 3052 (8.1%) |
| **Characteristics of Index COVID "Acute Phase"** |  |  |  |  |
| COVID Diagnosis during COVID-associated Hospitalization | 1757 (23.4%) | 4136 (11.0%) | 4135 (11.0%) | 3971 (10.6%) |
| COVID-associated Hospitalization | 2683 (35.7%) | 5382 (14.3%) | 5680 (15.1%) | 5436 (14.5%) |
| COVID-associated ED Visit | 1479 (19.7%) | 5858 (15.6%) | 5412 (14.4%) | 5264 (14.0%) |
| Hospitalization stay |  |  |  |  |
| Short Stay (0-2 days) | 560 (7.5%) | 1860 (5.0%) | 2006 (5.3%) | 1834 (4.9%) |
| Medium Stay (3-7 days) | 792 (10.5%) | 1966 (5.2%) | 2013 (5.4%) | 1974 (5.3%) |
| Long Stay (8-30 days) | 854 (11.4%) | 1137 (3.0%) | 1193 (3.2%) | 1187 (3.2%) |
| Extended Stay (31+ days) | 384 (5.1%) | 228 (0.6%) | 227 (0.6%) | 175 (0.5%) |
| COVID treatment |  |  |  |  |
| Corticosteroids^a^ | 1841 (24.5%) | 2752 (7.3%) | 2667 (7.1%) | 2636 (7.0%) |
| Remdisivir^a^ | 1261 (16.8%) | 1700 (4.5%) | 1585 (4.2%) | 1584 (4.2%) |
| Vasopressors^a^ | 464 (6.2%) | 617 (1.6%) | 674 (1.8%) | 659 (1.8%) |
| ECMO^a^ | 56 (0.7%) | 29 (0.1%) | 21 (0.1%) | <20 |
| Mechanical Ventilation^a^ | 528 (7.0%) | 434 (1.2%) | 413 (1.1%) | 369 (1.0%) |
| AKI during COVID-associated Hospitalization | 623 (8.3%) | 929 (2.5%) | 974 (2.6%) | 944 (2.5%) |
| Sepsis during COVID-associated Hospitalization | 580 (7.7%) | 796 (2.1%) | 761 (2.0%) | 793 (2.1%) |

^a^Only captured for individuals hospitalized for COVID-19

**eTable 15. PASC Risk Factors from Logistic Regression (PASC defined as U09.9)**

|  | Method 1  Unrestricted Controls  (N=45090) | Method 2  Restricted  controls  (N=45072) | Method 3  Most Restricted  controls  (N=45072) |
| --- | --- | --- | --- |
| **Demographics** |  |  |  |
| Age |  |  |  |
| 18-29 | REF | REF | REF |
| 30-39 | 1.76 (1.58-1.96) | 1.91 (1.71-2.13) | 1.84 (1.65-2.05) |
| 40-49 | 2.46 (2.21-2.73) | 2.95 (2.66-3.28) | 2.62 (2.36-2.92) |
| 50-59 | 2.62 (2.36-2.92) | 3.09 (2.78-3.44) | 2.78 (2.5-3.09) |
| 60-69 | 2.31 (2.06-2.59) | 2.87 (2.57-3.22) | 2.4 (2.15-2.69) |
| 70-79 | 1.87 (1.64-2.14) | 2.24 (1.96-2.55) | 1.97 (1.73-2.25) |
| 80-89 | 1.59 (1.33-1.91) | 1.83 (1.54-2.18) | 1.63 (1.36-1.95) |
| 90+ | 1.24 (0.86-1.78) | 1.26 (0.89-1.79) | 1.19 (0.83-1.7) |
| Sex |  |  |  |
| Male or Unknown Sex | REF | REF | REF |
| Female | 1.38 (1.3-1.46) | 1.32 (1.24-1.39) | 1.25 (1.18-1.32) |
| Race/ethnicity |  |  |  |
| White NH | REF | REF | REF |
| Hispanic | 0.76 (0.69-0.83) | 0.83 (0.75-0.91) | 0.86 (0.78-0.94) |
| Black NH | 0.75 (0.7-0.82) | 0.78 (0.72-0.84) | 0.81 (0.75-0.88) |
| Asian NH | 0.83 (0.67-1.02) | 0.75 (0.61-0.93) | 0.79 (0.64-0.98) |
| Other race NH | 0.92 (0.67-1.27) | 0.95 (0.69-1.31) | 1.0 (0.73-1.38) |
| Unknown | 0.73 (0.63-0.83) | 1.04 (0.9-1.19) | 1.0 (0.87-1.15) |
| **Comorbidities Prior to COVID Index Date** |  |  |  |
| AKI | 0.93 (0.79-1.09) | 0.91 (0.78-1.07) | 0.84 (0.71-0.98) |
| Cardiomyopathies | 0.92 (0.76-1.11) | 0.83 (0.7-1.0) | 0.85 (0.71-1.02) |
| Cerebrovascular Disease | 0.88 (0.76-1.01) | 0.84 (0.73-0.96) | 0.86 (0.75-0.98) |
| Chronic Lung Disease | 1.67 (1.56-1.79) | 1.57 (1.47-1.67) | 1.6 (1.5-1.7) |
| Complicated Diabetes | 1.03 (0.9-1.17) | 1.07 (0.94-1.21) | 1.0 (0.88-1.14) |
| Congestive Heart Failure | 0.87 (0.68-1.11) | 0.86 (0.68-1.08) | 0.87 (0.69-1.1) |
| Coronary Artery Disease | 1.02 (0.91-1.15) | 0.97 (0.87-1.08) | 0.96 (0.86-1.07) |
| Dementia | 0.84 (0.68-1.03) | 0.79 (0.65-0.96) | 0.88 (0.72-1.08) |
| Down Syndrome | 7.35 (0.73-73.97) | 4.45 (0.66-30.12) | 11.54 (1.14-116.28) |
| Heart Failure | 1.16 (0.93-1.44) | 1.24 (1.01-1.54) | 1.15 (0.93-1.42) |
| Hemiplegia or Paraplegia | 0.66 (0.48-0.9) | 0.55 (0.4-0.74) | 0.64 (0.47-0.88) |
| HIV | 0.85 (0.59-1.23) | 0.9 (0.63-1.28) | 0.74 (0.52-1.05) |
| Hypertension | 1.16 (1.09-1.25) | 0.96 (0.9-1.03) | 0.98 (0.91-1.04) |
| Kidney Disease | 1.01 (0.89-1.15) | 0.99 (0.87-1.13) | 1.04 (0.92-1.18) |
| Malignant Cancer | 1.06 (0.96-1.17) | 0.87 (0.79-0.96) | 0.91 (0.83-1.0) |
| Metastatic Solid Tumor Cancers | 0.68 (0.52-0.89) | 0.65 (0.51-0.84) | 0.64 (0.49-0.82) |
| Mild Liver Disease | 0.91 (0.74-1.13) | 0.9 (0.73-1.1) | 0.91 (0.74-1.12) |
| Moderate to Severe Liver Disease | 0.74 (0.54-1.01) | 0.7 (0.52-0.95) | 0.67 (0.49-0.9) |
| Myocardial Infarction | 0.76 (0.65-0.88) | 0.78 (0.67-0.9) | 0.85 (0.74-0.99) |
| Obesity | 1.27 (1.21-1.35) | 1.05 (0.99-1.11) | 1.07 (1.01-1.14) |
| Peptic Ulcer | 1.16 (0.99-1.36) | 1.14 (0.98-1.32) | 1.11 (0.96-1.29) |
| Perpheral Vascular Disease | 1.24 (1.07-1.43) | 1.1 (0.96-1.26) | 1.14 (0.99-1.31) |
| Rheumatologic Disease | 1.29 (1.11-1.49) | 1.24 (1.08-1.42) | 1.18 (1.03-1.36) |
| Sickle Cell Disease | 1.44 (0.76-2.72) | 0.85 (0.48-1.52) | 0.76 (0.42-1.36) |
| Systemic Corticosteroids | 1.35 (1.28-1.44) | 1.16 (1.1-1.23) | 1.19 (1.12-1.26) |
| Thalassemia | 1.69 (0.95-2.99) | 1.07 (0.63-1.8) | 1.22 (0.72-2.07) |
| Tuberculosis | 1.19 (0.72-1.95) | 1.12 (0.7-1.77) | 1.48 (0.92-2.36) |
| Uncomplicated Diabetes | 0.85 (0.76-0.95) | 0.83 (0.74-0.92) | 0.84 (0.76-0.94) |
| **Behavioral Health** |  |  |  |
| Depression | 1.55 (1.45-1.66) | 1.32 (1.24-1.41) | 1.28 (1.2-1.37) |
| Psychosis | 0.6 (0.45-0.81) | 0.58 (0.44-0.78) | 0.56 (0.42-0.76) |
| Substance Use | 0.66 (0.55-0.78) | 0.55 (0.47-0.65) | 0.65 (0.55-0.76) |
| Tobacco Smoker | 0.74 (0.66-0.83) | 0.7 (0.63-0.78) | 0.69 (0.62-0.76) |
| **Characteristics of Index COVID "Acute Phase"** |  |  |  |
| COVID Diagnosis during COVID-associated Hospitalization | 0.5 (0.45-0.57) | 0.65 (0.58-0.73) | 0.65 (0.58-0.73) |
| COVID-associated Hospitalization | 3.47 (2.66-4.54) | 2.85 (2.21-3.68) | 2.55 (1.99-3.27) |
| COVID-associated ED Visit | 1.27 (1.18-1.36) | 1.48 (1.38-1.59) | 1.51 (1.41-1.62) |
| Hospitalization stay |  |  |  |
| Not Hospitalized | REF | REF | REF |
| Short Stay | 0.79 (0.59-1.05) | 0.76 (0.58-1.0) | 0.96 (0.73-1.25) |
| Medium Stay | 0.81 (0.6-1.09) | 0.79 (0.6-1.05) | 1.0 (0.76-1.31) |
| Long Stay | 1.52 (1.13-2.05) | 1.42 (1.06-1.89) | 1.76 (1.33-2.34) |
| Extended Stay | 2.85 (1.97-4.11) | 2.52 (1.76-3.61) | 4.34 (3.02-6.25) |
| COVID treatment |  |  |  |
| Corticosteroidsᵃ | 1.19 (1.05-1.36) | 1.42 (1.25-1.61) | 1.37 (1.21-1.55) |
| Remdisivirᵃ | 1.38 (1.22-1.56) | 1.51 (1.34-1.71) | 1.43 (1.27-1.62) |
| Vasopressorsᵃ | 0.7 (0.58-0.84) | 0.67 (0.57-0.8) | 0.64 (0.54-0.77) |
| ECMOᵃ | 1.5 (0.91-2.47) | 2.04 (1.17-3.55) | 2.15 (1.18-3.89) |
| Medical Ventilationᵃ | 1.5 (1.22-1.84) | 1.61 (1.32-1.98) | 1.66 (1.35-2.04) |
| AKI during COVID-associated Hospitalization | 1.06 (0.91-1.25) | 1.14 (0.97-1.32) | 1.16 (0.99-1.35) |
| Sepsis during COVID-associated Hospitalization | 0.92 (0.8-1.07) | 0.98 (0.84-1.13) | 0.84 (0.73-0.98) |

ᵃOnly captured for individuals hospitalized for COVID-19

Odds ratios presented with 95% CI in parenthesis

**eTable 16. Characteristics of PASC defined as Long-COVID Clinic Visits**

|  | Long-COVID Clinic Visit Only  (N=1241) | Method 1  Unrestricted controls (N=6205) | Method 2  Restricted controls (N=6205) | Method 3  Most restricted controls (N=6205) |
| --- | --- | --- | --- | --- |
| **Demographics** |  |  |  |  |
| Age |  |  |  |  |
| 18-29 | 86 (6.9%) | 1105 (17.8%) | 1123 (18.1%) | 1155 (18.6%) |
| 30-39 | 211 (17.0%) | 1110 (17.9%) | 1025 (16.5%) | 1027 (16.6%) |
| 40-49 | 274 (22.1%) | 1109 (17.9%) | 1055 (17.0%) | 980 (15.8%) |
| 50-59 | 307 (24.7%) | 1094 (17.6%) | 1053 (17.0%) | 1069 (17.2%) |
| 60-69 | 230 (18.5%) | 907 (14.6%) | 971 (15.6%) | 969 (15.6%) |
| 70-79 | 104 (8.4%) | 580 (9.3%) | 613 (9.9%) | 610 (9.8%) |
| 80-89 | 29 (2.3%) | 236 (3.8%) | 287 (4.6%) | 308 (5.0%) |
| 90+ | 0 (0.0%) | 64 (1.0%) | 78 (1.3%) | 87 (1.4%) |
| Sex |  |  |  |  |
| Female | 776 (62.5%) | 3438 (55.4%) | 3642 (58.7%) | 3688 (59.4%) |
| Male | 464 (37.4%) | 2757 (44.4%) | 2561 (41.3%) | 2512 (40.5%) |
| Race/ethnicity |  |  |  |  |
| White non-Hispanic (NH) | 699 (56.3%) | 3482 (56.1%) | 3618 (58.3%) | 3665 (59.1%) |
| Hispanic | 176 (14.2%) | 948 (15.3%) | 823 (13.3%) | 850 (13.7%) |
| Black NH | 267 (21.5%) | 1181 (19.0%) | 1334 (21.5%) | 1254 (20.2%) |
| Asian NH | 26 (2.1%) | 188 (3.0%) | 156 (2.5%) | 171 (2.8%) |
| Other race NH | <20 | <20 | <20 | 22 (0.4%) |
| Unknown | 70 (5.6%) | 387 (6.2%) | 252 (4.1%) | 239 (3.9%) |
| **Comorbidities Prior to COVID Index Date** |  |  |  |  |
| AKI | 71 (5.7%) | 278 (4.5%) | 301 (4.9%) | 290 (4.7%) |
| Cardiomyopathies | 32 (2.6%) | 97 (1.6%) | 141 (2.3%) | 112 (1.8%) |
| Cerebrovascular Disease | 35 (2.8%) | 164 (2.6%) | 204 (3.3%) | 196 (3.2%) |
| Chronic Lung Disease | 275 (22.2%) | 731 (11.8%) | 896 (14.4%) | 799 (12.9%) |
| Complicated Diabetes | 126 (10.2%) | 474 (7.6%) | 598 (9.6%) | 535 (8.6%) |
| Congestive Heart Failure | 76 (6.1%) | 194 (3.1%) | 261 (4.2%) | 231 (3.7%) |
| Coronary Artery Disease | 90 (7.3%) | 336 (5.4%) | 423 (6.8%) | 400 (6.4%) |
| Dementia | <20 | 83 (1.3%) | 125 (2.0%) | 128 (2.1%) |
| Down Syndrome | 0 (0.0%) | <20 | 0 (0.0%) | 0 (0.0%) |
| Heart Failure | 97 (7.8%) | 259 (4.2%) | 341 (5.5%) | 302 (4.9%) |
| Hemiplegia or Paraplegia | <20 | 28 (0.5%) | 36 (0.6%) | 37 (0.6%) |
| HIV | <20 | 32 (0.5%) | 40 (0.6%) | 34 (0.5%) |
| Hypertension | 371 (29.9%) | 1446 (23.3%) | 1780 (28.7%) | 1710 (27.6%) |
| Kidney Disease | 119 (9.6%) | 450 (7.3%) | 566 (9.1%) | 512 (8.3%) |
| Malignant Cancer | 91 (7.3%) | 444 (7.2%) | 536 (8.6%) | 485 (7.8%) |
| Metastatic Solid Tumor Cancers | <20 | 60 (1.0%) | 73 (1.2%) | 69 (1.1%) |
| Mild Liver Disease | <20 | 61 (1.0%) | 99 (1.6%) | 75 (1.2%) |
| Moderate to Severe Liver Disease | <20 | 25 (0.4%) | 40 (0.6%) | 29 (0.5%) |
| Myocardial Infarction | 32 (2.6%) | 169 (2.7%) | 213 (3.4%) | 194 (3.1%) |
| Obesity | 643 (51.8%) | 2418 (39.0%) | 2923 (47.1%) | 2798 (45.1%) |
| Peptic Ulcer | 21 (1.7%) | 79 (1.3%) | 114 (1.8%) | 99 (1.6%) |
| Perpheral Vascular Disease | 42 (3.4%) | 149 (2.4%) | 197 (3.2%) | 170 (2.7%) |
| Rheumatologic Disease | 33 (2.7%) | 108 (1.7%) | 138 (2.2%) | 113 (1.8%) |
| Sickle Cell Disease | <20 | <20 | <20 | <20 |
| Systemic Corticosteroids | 491 (39.6%) | 1816 (29.3%) | 2147 (34.6%) | 2010 (32.4%) |
| Thalassemia | <20 | <20 | <20 | <20 |
| Tuberculosis | <20 | <20 | <20 | <20 |
| Uncomplicated Diabetes | 189 (15.2%) | 740 (11.9%) | 898 (14.5%) | 856 (13.8%) |
| **Behavioral Health** |  |  |  |  |
| Depression | 218 (17.6%) | 630 (10.2%) | 844 (13.6%) | 780 (12.6%) |
| Psychosis | <20 | 47 (0.8%) | 62 (1.0%) | 55 (0.9%) |
| Substance Abuse | <20 | 145 (2.3%) | 171 (2.8%) | 165 (2.7%) |
| Tobacco Smoker | 21 (1.7%) | 269 (4.3%) | 348 (5.6%) | 297 (4.8%) |
| **Characteristics of Index COVID "Acute Phase"** |  |  |  |  |
| COVID Diagnosis during COVID-associated Hospitalization | 394 (31.7%) | 754 (12.2%) | 793 (12.8%) | 756 (12.2%) |
| COVID-associated Hospitalization | 547 (44.1%) | 1089 (17.6%) | 1167 (18.8%) | 1085 (17.5%) |
| COVID-associated ED Visit | 167 (13.5%) | 1098 (17.7%) | 978 (15.8%) | 982 (15.8%) |
| Hospitalization Stay |  |  |  |  |
| Short Stay (0-2 days) | 76 (6.1%) | 383 (6.2%) | 404 (6.5%) | 374 (6.0%) |
| Medium Stay (3-7 days) | 106 (8.5%) | 369 (5.9%) | 395 (6.4%) | 352 (5.7%) |
| Long Stay (8-30 days) | 220 (17.7%) | 214 (3.4%) | 215 (3.5%) | 228 (3.7%) |
| Extended Stay (31+ days) | 86 (6.9%) | 32 (0.5%) | 52 (0.8%) | 46 (0.7%) |
| COVID Treatment |  |  |  |  |
| Corticosteroids^a^ | 272 (21.9%) | 475 (7.7%) | 503 (8.1%) | 463 (7.5%) |
| Remdisivir^a^ | 217 (17.5%) | 335 (5.4%) | 325 (5.2%) | 300 (4.8%) |
| Vasopressors^a^ | 170 (13.7%) | 114 (1.8%) | 149 (2.4%) | 132 (2.1%) |
| ECMO^a^ | <20 | <20 | <20 | <20 |
| Mechanical Ventilation^a^ | 121 (9.8%) | 64 (1.0%) | 74 (1.2%) | 69 (1.1%) |
| AKI during COVID-associated Hospitalization | 67 (5.4%) | 122 (2.0%) | 145 (2.3%) | 119 (1.9%) |
| Sepsis during COVID-associated Hospitalization | 68 (5.5%) | 92 (1.5%) | 121 (2.0%) | 102 (1.6%) |

**eTable 17. PASC Risk Factors from Logistic Regression (PASC defined as Long-COVID**

**Clinic Visits)**

|  | Method 1  Unrestricted Controls  (N=7446) | Method 2  Restricted  controls  (N=7446) | Method 3  Most Restricted  controls  (N=7446) |
| --- | --- | --- | --- |
| **Demographics** |  |  |  |
| Age |  |  |  |
| 18-29 | REF | REF | REF |
| 30-39 | 2.24 (1.7-2.95) | 2.6 (1.97-3.42) | 2.69 (2.04-3.55) |
| 40-49 | 2.63 (2.01-3.45) | 3.14 (2.4-4.1) | 3.54 (2.7-4.64) |
| 50-59 | 2.57 (1.96-3.37) | 3.24 (2.47-4.26) | 3.21 (2.44-4.21) |
| 60-69 | 1.94 (1.45-2.6) | 2.3 (1.72-3.07) | 2.11 (1.58-2.83) |
| 70-79 | 1.22 (0.86-1.72) | 1.48 (1.05-2.08) | 1.5 (1.07-2.1) |
| 80-89 | 0.69 (0.41-1.15) | 0.6 (0.36-1.0) | 0.54 (0.33-0.9) |
| 90+ | 0.0 (0.0-inf) | 0.0 (0.0-inf) | 0.0 (0.0-inf) |
| Sex |  |  |  |
| Male or Unknown Sex | REF | REF | REF |
| Female | 1.49 (1.29-1.72) | 1.35 (1.17-1.56) | 1.23 (1.07-1.42) |
| Race/ethnicity |  |  |  |
| White NH | REF | REF | REF |
| Hispanic | 0.67 (0.55-0.83) | 0.77 (0.62-0.95) | 0.73 (0.59-0.91) |
| Black NH | 0.77 (0.64-0.92) | 0.68 (0.57-0.82) | 0.74 (0.62-0.89) |
| Asian NH | 0.56 (0.36-0.89) | 0.62 (0.39-0.99) | 0.59 (0.37-0.93) |
| Other race NH | 0.75 (0.15-3.68) | 0.5 (0.09-2.65) | 0.34 (0.07-1.59) |
| Unknown | 0.94 (0.7-1.26) | 1.25 (0.92-1.7) | 1.39 (1.02-1.9) |
| **Comorbidities Prior to COVID Index Date** |  |  |  |
| AKI | 0.49 (0.28-0.84) | 0.72 (0.42-1.21) | 0.59 (0.34-1.02) |
| Cardiomyopathies | 1.19 (0.7-2.04) | 0.9 (0.55-1.48) | 1.01 (0.61-1.67) |
| Cerebrovascular Disease | 0.71 (0.45-1.11) | 0.87 (0.56-1.34) | 0.89 (0.57-1.38) |
| Chronic Lung Disease | 1.48 (1.22-1.79) | 1.52 (1.26-1.83) | 1.67 (1.38-2.02) |
| Complicated Diabetes | 0.87 (0.6-1.26) | 0.79 (0.55-1.14) | 0.92 (0.63-1.33) |
| Congestive Heart Failure | 1.6 (0.8-3.17) | 1.32 (0.68-2.55) | 1.68 (0.85-3.31) |
| Coronary Artery Disease | 1.67 (1.21-2.31) | 1.56 (1.14-2.15) | 1.41 (1.03-1.94) |
| Dementia | 0.98 (0.54-1.8) | 0.95 (0.53-1.71) | 0.83 (0.46-1.51) |
| Heart Failure | 0.77 (0.42-1.43) | 0.98 (0.54-1.77) | 0.87 (0.47-1.61) |
| Hemiplegia or Paraplegia | 0.84 (0.3-2.33) | 0.71 (0.28-1.81) | 0.53 (0.2-1.37) |
| HIV | 1.58 (0.73-3.41) | 1.7 (0.83-3.47) | 1.61 (0.78-3.34) |
| Hypertension | 1.14 (0.94-1.37) | 1.0 (0.84-1.2) | 0.99 (0.82-1.18) |
| Kidney Disease | 1.06 (0.72-1.57) | 0.87 (0.6-1.25) | 0.87 (0.6-1.27) |
| Malignant Cancer | 0.89 (0.67-1.19) | 0.82 (0.62-1.07) | 0.9 (0.69-1.19) |
| Metastatic Solid Tumor Cancers | 0.42 (0.17-1.01) | 0.44 (0.19-1.07) | 0.5 (0.21-1.18) |
| Mild Liver Disease | 1.13 (0.58-2.22) | 0.76 (0.41-1.42) | 0.89 (0.47-1.68) |
| Moderate to Severe Liver Disease | 0.57 (0.2-1.62) | 0.57 (0.21-1.51) | 0.83 (0.29-2.4) |
| Myocardial Infarction | 0.44 (0.27-0.73) | 0.46 (0.28-0.73) | 0.5 (0.31-0.81) |
| Obesity | 1.07 (0.92-1.25) | 0.86 (0.74-1.0) | 0.91 (0.78-1.05) |
| Peptic Ulcer | 1.0 (0.57-1.75) | 1.07 (0.63-1.79) | 0.93 (0.54-1.6) |
| Perpheral Vascular Disease | 1.29 (0.83-2.03) | 1.19 (0.78-1.83) | 1.46 (0.95-2.27) |
| Rheumatologic Disease | 1.02 (0.65-1.59) | 0.94 (0.61-1.46) | 1.51 (0.97-2.35) |
| Sickle Cell Disease | 0.88 (0.2-3.86) | 0.55 (0.1-2.92) | 1.56 (0.31-7.95) |
| Systemic Corticosteroids | 1.11 (0.95-1.31) | 0.99 (0.85-1.16) | 1.05 (0.9-1.23) |
| Thalassemia | 1.46 (0.36-5.85) | 0.78 (0.17-3.56) | 4.69 (1.05-21.01) |
| Tuberculosis | 1.57 (0.47-5.26) | 3.42 (1.02-11.46) | 1.44 (0.47-4.44) |
| Uncomplicated Diabetes | 0.79 (0.58-1.07) | 0.86 (0.64-1.16) | 0.77 (0.57-1.03) |
| **Behavioral Health** |  |  |  |
| Depression | 1.82 (1.49-2.22) | 1.52 (1.25-1.84) | 1.61 (1.33-1.95) |
| Psychosis | 0.4 (0.16-1.04) | 0.25 (0.1-0.65) | 0.4 (0.15-1.05) |
| Substance Abuse | 0.6 (0.34-1.04) | 0.66 (0.39-1.13) | 0.48 (0.28-0.84) |
| Tobacco Smoker | 0.27 (0.16-0.45) | 0.27 (0.17-0.44) | 0.32 (0.2-0.52) |
| **Characteristics of Index COVID "Acute Phase"** |  |  |  |
| COVID Diagnosis during COVID-associated Hospitalization | 1.0 (0.76-1.32) | 1.13 (0.86-1.49) | 1.01 (0.76-1.34) |
| COVID-associated Hospitalization | 5.05 (3.48-7.33) | 4.58 (3.19-6.58) | 6.13 (4.2-8.93) |
| COVID-associated ED Visit | 0.85 (0.7-1.03) | 1.01 (0.83-1.23) | 0.98 (0.81-1.2) |
| Hospitalization stay |  |  |  |
| Not Hospitalized | REF | REF | REF |
| Short Stay | 0.34 (0.22-0.54) | 0.35 (0.22-0.54) | 0.29 (0.18-0.46) |
| Medium Stay | 0.55 (0.35-0.88) | 0.56 (0.36-0.88) | 0.48 (0.3-0.76) |
| Long Stay | 2.16 (1.35-3.47) | 2.26 (1.43-3.61) | 1.74 (1.08-2.82) |
| Extended Stay | 4.05 (2.02-8.14) | 2.43 (1.24-4.74) | 2.68 (1.35-5.33) |
| COVID treatment |  |  |  |
| Corticosteroidsᵃ | 0.76 (0.55-1.04) | 0.76 (0.56-1.03) | 0.67 (0.49-0.92) |
| Remdesivirᵃ | 1.15 (0.83-1.61) | 1.35 (0.98-1.86) | 1.73 (1.25-2.4) |
| Vasopressorsᵃ | 1.51 (1.0-2.29) | 1.39 (0.94-2.04) | 1.64 (1.09-2.47) |
| ECMOᵃ | 1.81 (0.55-5.92) | 2.42 (0.76-7.77) | 1.82 (0.61-5.46) |
| Mechanical Ventilationᵃ | 1.38 (0.83-2.3) | 1.43 (0.87-2.36) | 0.99 (059-1.64) |
| AKI during COVID-associated Hospitalization | 0.95 (0.56-1.6) | 1.09 (0.66-1.8) | 1.09 (0.65-1.85) |
| Sepsis during COVID-associated Hospitalization | 0.64 (0.41-0.99) | 0.49 (0.32-0.75) | 0.55 (0.36-0.85) |

ᵃOnly captured individuals hospitalized for COVID-19

Odds ratios presented with 95% CI in parenthesis

**eTable 18. Comparison of U09.9 and Long-COVID Visit Cohorts**

|  | U09.9 OR Long-COVID Visit (N=8325) | U09.9 Only (N=7512) | Long-COVID Clinic Visit Only (N=1241) |
| --- | --- | --- | --- |
| **Demographics** |  |  |  |
| Age |  |  |  |
| 18-29 | 630 (7.6%) | 577 (7.7%) | 86 (6.9%) |
| 30-39 | 1229 (14.8%) | 1090 (14.5%) | 211 (17.0%) |
| 40-49 | 1749 (21.0%) | 1580 (21.0%) | 274 (22.1%) |
| 50-59 | 1933 (23.2%) | 1738 (23.1%) | 307 (24.7%) |
| 60-69 | 1605 (19.3%) | 1450 (19.3%) | 230 (18.5%) |
| 70-79 | 840 (10.1%) | 766 (10.2%) | 104 (8.4%) |
| 80-89 | 293 (3.5%) | 268 (3.6%) | 29 (2.3%) |
| 90+ | 37 (0.4%) | 43 (0.6%) | 0 (0.0%) |
| Sex |  |  |  |
| Female | 5225 (62.8%) | 4752 (63.3%) | 776 (62.5%) |
| Male | 3096 (37.2%) | 2759 (36.7%) | 464 (37.4%) |
| Race/ethnicity |  |  |  |
| White non-Hispanic (NH) | 5707 (68.6%) | 5301 (70.6%) | 699 (56.3%) |
| Hispanic | 835 (10.0%) | 711 (9.5%) | 176 (14.2%) |
| Black NH | 1235 (14.8%) | 1035 (13.8%) | 267 (21.5%) |
| Asian NH | 136 (1.6%) | 116 (1.5%) | 26 (2.1%) |
| Other race NH | 54 (0.6%) | 52 (0.7%) | <20 |
| Unknown | 340 (4.1%) | 282 (3.8%) | 70 (5.6%) |
| **Comorbidities Prior to COVID Index Date** |  |  |  |
| AKI | 862 (10.4%) | 814 (10.8%) | 71 (5.7%) |
| Cardiomyopathies | 225 (2.7%) | 208 (2.8%) | 32 (2.6%) |
| Cerebrovascular Disease | 390 (4.7%) | 364 (4.8%) | 35 (2.8%) |
| Chronic Lung Disease | 2404 (28.9%) | 2245 (29.9%) | 275 (22.2%) |
| Complicated Diabetes | 1210 (14.5%) | 1136 (15.1%) | 126 (10.2%) |
| Congestive Heart Failure | 573 (6.9%) | 522 (6.9%) | 76 (6.1%) |
| Coronary Artery Disease | 832 (10.0%) | 778 (10.4%) | 90 (7.3%) |
| Dementia | 153 (1.8%) | 145 (1.9%) | <20 |
| Down Syndrome | <20 | <20 | 0 (0.0%) |
| Heart Failure | 737 (8.9%) | 669 (8.9%) | 97 (7.8%) |
| Hemiplegia or Paraplegia | 61 (0.7%) | 58 (0.8%) | <20 |
| HIV | 51 (0.6%) | 41 (0.5%) | <20 |
| Hypertension | 3365 (40.4%) | 3145 (41.9%) | 371 (29.9%) |
| Kidney Disease | 1262 (15.2%) | 1183 (15.7%) | 119 (9.6%) |
| Malignant Cancer | 837 (10.1%) | 783 (10.4%) | 91 (7.3%) |
| Metastatic Solid Tumor Cancers | 91 (1.1%) | 87 (1.2%) | <20 |
| Mild Liver Disease | 170 (2.0%) | 159 (2.1%) | <20 |
| Moderate to Severe Liver Disease | 82 (1.0%) | 77 (1.0%) | <20 |
| Myocardial Infarction | 392 (4.7%) | 371 (4.9%) | 32 (2.6%) |
| Obesity | 4691 (56.4%) | 4279 (57.0%) | 643 (51.8%) |
| Peptic Ulcer | 279 (3.4%) | 270 (3.6%) | 21 (1.7%) |
| Perpheral Vascular Disease | 405 (4.9%) | 388 (5.2%) | 42 (3.4%) |
| Rheumatologic Disease | 350 (4.2%) | 334 (4.4%) | 33 (2.7%) |
| Sickle Cell Disease | <20 | <20 | <20 |
| Systemic Corticosteroids | 4325 (52.0%) | 4015 (53.4%) | 491 (39.6%) |
| Thalassemia | 21 (0.3%) | <20 | <20 |
| Tuberculosis | 27 (0.3%) | 24 (0.3%) | <20 |
| Uncomplicated Diabetes | 1708 (20.5%) | 1589 (21.2%) | 189 (15.2%) |
| **Behavioral Health** |  |  |  |
| Depression | 2059 (24.7%) | 1946 (25.9%) | 218 (17.6%) |
| Psychosis | 65 (0.8%) | 61 (0.8%) | <20 |
| Substance Abuse | 205 (2.5%) | 196 (2.6%) | <20 |
| Tobacco Smoker | 515 (6.2%) | 504 (6.7%) | 21 (1.7%) |
| **Characteristics of Index COVID "Acute Phase"** |  |  |  |
| COVID Diagnosis during COVID-associated Hospitalization | 2065 (24.8%) | 1757 (23.4%) | 394 (31.7%) |
| COVID-associated Hospitalization | 3100 (37.3%) | 2683 (35.7%) | 547 (44.1%) |
| COVID-associated ED Visit | 1564 (18.8%) | 1479 (19.7%) | 167 (13.5%) |
| Hospitalization Stay |  |  |  |
| Short Stay | 610 (7.3%) | 560 (7.5%) | 76 (6.1%) |
| Medium Stay | 870 (10.5%) | 792 (10.5%) | 106 (8.5%) |
| Long Stay | 1029 (12.4%) | 854 (11.4%) | 220 (17.7%) |
| Extended Stay | 449 (5.4%) | 384 (5.1%) | 86 (6.9%) |
| COVID treatment |  |  |  |
| Corticosteroidsᵃ | 2025 (24.3%) | 1841 (24.5%) | 272 (21.9%) |
| Remdisivirᵃ | 1409 (16.9%) | 1261 (16.8%) | 217 (17.5%) |
| Vasopressorsᵃ | 601 (7.2%) | 464 (6.2%) | 170 (13.7%) |
| ECMOᵃ | 66 (0.8%) | 56 (0.7%) | <20 |
| Mechanical Ventilationᵃ | 615 (7.4%) | 528 (7.0%) | 121 (9.8%) |
| AKI during COVID-associated Hospitalization | 664 (8.0%) | 623 (8.3%) | 67 (5.4%) |
| Sepsis during COVID-associated Hospitalization | 614 (7.4%) | 580 (7.7%) | 68 (5.5%) |

ᵃOnly captured for individuals hospitalized with COVID-19
